# Genomic characterization of foodborne *Salmonella enterica* and *Escherichia coli* isolates from Saboba district and Bolgatanga Municipality Ghana

**DOI:** 10.1371/journal.pone.0315583

**Published:** 2025-02-07

**Authors:** Gabriel Temitope Sunmonu, Frederick Adzitey, Erkison Ewomazino Odih, Boniface Awini Tibile, Rejoice Ekli, Martin Aduah, Anderson O. Oaikhena, Olabisi C. Akinlabi, Akebe Luther King Abia, Daniel Gyamfi Amoako, Iruka N. Okeke

**Affiliations:** 1 Department of Pharmaceutical Microbiology, Faculty of Pharmacy, University of Ibadan, Ibadan, Nigeria; 2 Faculty of Agriculture, Food and Consumer Sciences, University for Development Studies, Tamale, Ghana; 3 Antimicrobial Research Unit, College of Health Sciences, University of KwaZulu-Natal, Durban, South Africa; 4 Department of Integrative Biology and Bioinformatics, University of Guelph, Guelph, Ontario, Canada; North Carolina State University, UNITED STATES OF AMERICA

## Abstract

*Salmonella enterica* and *Escherichia coli* are well-known bacteria commonly associated with foodborne illnesses in humans and animals. Genomic characterization of these pathogens provides valuable insights into their evolution, virulence factors and resistance determinants. This study aimed to characterized previously isolated *Salmonella* (n = 14) and *E*. *coli* (n = 19) from milk, meat and its associated utensils in Ghana using whole-genome sequencing. Most of the *Salmonella* serovars (Fresno, Plymouth, Infantis, Give and Orleans) identified in this study are yet to be reported in Ghana. Most *Salmonella* isolates were pan-sensitive, but genes conferring resistance to fosfomycin (*fosA7*.*2*) and tetracycline (*tet(A)*) were detected in one and three isolates, respectively. Seven of the *Salmonella* isolates carried the IncI1-I(Gamma) plasmid replicon. Although antimicrobial resistance was not common among *Salmonella* strains, most (11/19) of the *E*. *coli* strains had at least one resistance gene, with nearly half (8/19) being multidrug resistant and carried plasmids. Three of the 19 *E*. *coli* strains belonged to serovars commonly associated with enteroaggregative *E*. *coli* (EAEC) pathotype. While strains belonging to virulence-associated lineages lacked key plasmid-encoded virulence plasmids, several plasmid replicons were detected in most of the *E*. *coli* (14/19) strains. Food contaminated with these pathogens can serve as a vehicle for disease transmission, posing a significant public health risk and necessitating stringent food safety and hygiene practices to prevent outbreaks. Hence, there is need for continuous surveillance and preventive measures to stop the spread of foodborne pathogens and reduce the risk of associated illnesses in Ghana.

## Introduction

Milk and meat are essential protein sources that constitute a significant and nutrient-rich component of human diets. However, their consumption is often associated with foodborne infections [1], particularly those caused by *Salmonella* and *Escherichia coli* [2–5]. Salmonellosis is under-reported in Ghana, and only a few studies have investigated the plausible role of contaminated milk [6], meat, meat products, handlers’ hands and associated surfaces such as knives, tables and aprons [7–9] in facilitating their transmission. Knowledge of food safety practices by key food handlers in Ghana has recently been reported to be suboptimal, as have food safety infrastructure and regulatory enforcement [10, 11]. There have been a few reports of meat samples contaminated with *E*. *coli* in Ghana [11–14]. Similarly, *E*. *coli* has been recovered from milk, milking utensils, faeces of lactating cow and milkers’ hands [15]. Some of these *E*. *coli* strains harbour both virulence and antimicrobial resistance genes (ARGs), raising public health concerns. However, few of these strains have been thoroughly characterized.

There are public health and food safety implications of finding *Salmonella* and *E*. *coli* in food because they are invariably of faecal origin. Globally, in addition to contamination risks, *Salmonella* and *E*. *coli* sourced from milk and meat increasingly exhibit resistance to different classes of antibiotics that is commonly mediated by mobile elements [13, 16–18]. The prevalence of multidrug-resistant (MDR) *Salmonella* and *E*. *coli* is also on the rise in clinical infections [19, 20]. Notably, resistance to extended spectrum beta-lactams, trimethoprim/sulfamethoxazole, chloramphenicol and ciprofloxacin has been reported, often associated with plasmids that could mediate their spread, in both *Salmonella* and *E*. *coli* isolates from milk and retail meats in Ghana [6, 12, 14].

Various methods, including serotyping, antibiotic profiling, pulsed-field gel electrophoresis and whole genome sequencing, have been employed to elucidate the phenotypic and genotypic attributes of foodborne pathogens and to determine their interrelationships and connections to pandemic clones of interest [13, 21]. Next-generation sequencing (NGS) technology, the most versatile and informative approach, has gained recent prominence [22, 23]. NGS is now used by PulseNet to categorize foodborne diseases, enabling nuanced epidemiological investigations [24]. Identification of virulence factors, antibiotic resistance genes, and serotypes are all possible by genomic analysis, which can also provide enhanced information on strain inter-relatedness, and therefore enable source attribution. Data on the genomic characterization of *Salmonella* and *E*. *coli* from milk and meat are few from low- and middle-income countries, including Ghana [7, 25]. In light of this gap, this study aims to characterize the resistance, virulence and plasmid profile of previously isolated *Salmonella* and *E*. *coli* isolated from fresh different retail meats, milk, and associated samples (handler’s hand swab, table, knife and faecal samples) in Saboba district and Bolgatanga Municipality of Ghana.

## Methods

### Strains

A total of 33 bacterial isolates (14 *Salmonella* and 19 *E*. *coli* species) previously isolated from various fresh and ready-to-eat meats, meat sellers’ tables, milk, milk-collecting utensils, milkers’ hands and faeces of lactating cows were characterized for this study (Table 1) [8, 15, 26, 27]. The isolates originated from markets and farms in Bolgatanga Municipality and Saboba District in Northern Ghana and were cryopreserved in 50% glycerol in Luria broth at -80°C.

**Table 1:** *E*. *coli* and *Salmonella* isolates characterized in this study.

Id	Code	Collection date	Species	Sample	Ref-erence
GH-FA-M23_S31	M2 *3*	2/10/2020	*Salmonella enterica*	Milk	[27]
GH-FA-M87_S6	M8 *1*	2/10/2020	*Salmonella enterica*	Milk	[27]
GH-FA-FS24_S23	FS24	2/10/2020	*Salmonella enterica*	Faecal sample (Milking cow)	[27]
GH-FA-M25_S2	M25	2/10/2020	*Salmonella enterica*	Milk	[27]
GH-FA-US15_S32	US15-2	22/10/2020	*Salmonella enterica*	Utensil sample	[27]
GH-FA-FK4_S10	FK4	22/10/2020	*Salmonella enterica*	Knife (Fresh meat)	[8]
GH-FA-FK4d_S7	FK4d	22/10/2020	*Salmonella enterica*	Knife (Fresh meat)	[8]
GH-FA-RCH2_S11	Rch2 extra	22/10/2020	*Salmonella enterica*	RTE Chicken	[8]
GH-FA-RG2_S29	RG2	22/10/2020	*Salmonella enterica*	RTE Guinea fowl	[8]
GH-FA-RK4_S19	RK4	2/10/2020	*Salmonella enterica*	Knife (RTE utensil)	[8]
GH-FA-RM4_S20	RM4	2/10/2020	*Salmonella enterica*	RTE Mutton	[8]
GH-FA-RP5_S5	RP5	12/10/2020	*Salmonella enterica*	RTE Pork	[8]
GH-FA-RP5D_S11	RP5D	12/10/2020	*Salmonella enterica*	RTE Pork	[8]
GH-FA-RPSD_S30	RPSD	12/10/2020	*Salmonella enterica*	RTE Pork	[8]
GH-FA-FS23_S22	FS23	22/10/2020	*Escherichia coli*	Faecal sample (milking cow)	[27]
GH-FA-US24_S14	US24	22/10/2020	*Escherichia coli*	Utensil Sample	[15]
GH-FA-HS11_S26	HS11	22/10/2020	*Escherichia coli*	Hand swab	[15]
GH-FA-HS3_S27	HS3	2/10/2020	*Escherichia coli*	Hand swab	[15]
GH-FA-M9_S16	M9	22/10/2020	*Escherichia coli*	Milk	[15]
GH-FA-US3_S21	US3	2/10/2020	*Escherichia coli*	Utensil sample	[15]
GH-FA-HS12_S21	HS12	2/10/2020	*Escherichia coli*	Hand Swab	[15]
GH-FA-M25D_S22	M25	2/10/2020	*Escherichia coli*	Milk	[15]
GH-FA-FB4_S24	FB4	22/10/2020	*Escherichia coli*	Fresh Beef	[26)
GH-FA-FC1_S23	FC1	2/10/2020	*Escherichia coli*	Fresh Chicken	[26]
GH-FA-FH2_S15	FH2	22/10/2020	*Escherichia coli*	Hand Swab (Fresh meat vendor)	[26]
GH-FA-FGS_S16	FG5	22/10/2020	*Escherichia coli*	Fresh Guinea fowl	[26]
GH-FA-FM4_S34	FM4	12/10/2020	*Escherichia coli*	Fresh Mutton	[26]
GH-FA-FT1_S17	FT1	12/10/2020	*Escherichia coli*	Table swab (Fresh meat)	[26)
GH-FA-RB4_S24	RB4	12/10/2020	*Escherichia coli*	RTE Beef	[26]
GH-FA-RH4_S3	RH4	12/10/2020	*Escherichia coli*	Hand swab (RTE meat Vendor)	[26]
GH-FA-RT3_S6	RT3	12/10/2020	*Escherichia coli*	Table swab (RTE meat)	[26]
GH-FA-RT3_S29	RT3d	12/10/2020	*Escherichia coli*	Table swab (RTE meat)	[26]
GH-FA-RCLI_S18	Rch1	12/10/2020	*Escherichia coli*	RTE Chevon	[26]

Key: RTE: Ready-to-eat–meats sampled in prepared form

### Ethical considerations

All isolates were recovered in earlier studies from vended food or at informal food vending premises, including milk cow droppings [8, 15, 26, 27]. Study design and sampling was approved by the Department of Veterinary Science, UDS. No other permissions were obtained or deemed necessary by the department. No humans or animals were use in the research and therefore ethical approval was deemed not required.

### *Salmonella* and *E*. *coli* identification

*Salmonella* isolates were initially confirmed using a latex agglutination kit for *Salmonella* (Oxoid Limited, Basingstoke, UK) and by PCR targeting the *invA* gene as described by Rahn et al. (1992) [28], using PCR oligonucleotides *invA139f* GTGAAATTATCGCCACGTTCGGGCAA and *invA141r* TCATCGCACCGTCAAGGAACC. PCR was performed using PuRe Taq Ready-To-Go PCR Beads (illustra^TM^). The PCR cycle used an initial denaturation temperature of 95°C for two minutes, followed by 35 cycles of denaturation at 95°C for 30 seconds, annealing at 55°C for 30 seconds and extension at 72°C for two minutes, then a terminal extension at 72°C for five minutes. Visualization of the 284 bp amplicon was accomplished after electrophoresis on 1.5% (w/v) agarose gels stained with Gel red (biotium), using a UVP GelMax transilluminator and imager. *Salmonella* isolates positive for *invA* and *E*. *coli* isolates were biotyped with the Gram-negative (GN) test kit (Ref: 21341) on VITEK 2 systems (version 2.0, Marcy-l’Etoile, France, Biomérieux) according to manufacturer’s instructions

### DNA extraction, library preparation and whole genome sequencing

Genomic DNA of the isolates was extracted using Wizard DNA extraction kit (Promega; Wisconsin, USA) in accordance with manufacturer’s protocol. Using a dsDNA Broad Range quantification assay, the extracted DNA was quantified on a Qubit fluorometer (Invitrogen; California, USA). dsDNA libraries were prepared using NEBNext Ultra II FS DNA library kit for Illumina with 96-unique indexes (New England Biolabs, Massachusetts, USA; Cat. No: E6609L). DNA libraries was quantified using dsDNA High Sensitivity quantification assay on a Quibit fluorometer (Invitrogen; California, USA) and fragment length analysed with the Bioanalyzer (Agilent). Denatured libraries were sequenced on an Illumina MiSeq (Illumina, California, USA). The raw sequence reads were *de novo* assembled using SPAdes v3.15.3 [29] according to GHRU protocols (https://gitlab.com/cgps/ghru/pipelines/dsl2/pipelines/assembly).

### Sequence typing of *Salmonella* and *E*. *coli* genomes

Sequence reads were deposited in the *Salmonella* and *E*. *coli* database for *Salmonella* and *E*. *coli* respectively on EnteroBase [30] and analyzed using publicly available tools that we have previously validated [31, 32]. Multi-locus sequence types (MLST) for the isolates were determined using ARIBA [33]. Novel ST strains were assigned ST using EnteroBase [30]. The *Salmonella* genome assemblies were analysed using the *Salmonella* In-Silico Typing Resource (SISTR) for the prediction of serovars and serogroups [34] (https://github.com/phac-nml/sistr_cmd), while the serotyping of the *E*. *coli* genome was done using ECtyper [35].

### Identification of AMR, plasmids and virulence genes

PlasmidFinder [36] was utilized to identify plasmid replicons that were present in the assembled genomes. AMRFinderPlus v3.10.24 [37] and its associated database (version 2022-04-04.1) were used to predict the antimicrobial resistance genes carried by the isolates and the drug classes to which they probably conferred resistance. Using ARIBA [33] and the virulence factor database (VFDB, http://www.mgc.ac.cn/VFs/), we were also able to identify the virulence genes that were present in the isolates.

### Single Nucleotide Polymorphism (SNP) calling and phylogenetic analysis

For phylogenetic analysis, reference sequences for the *Salmonella* and *E*. *coli* genomes were objectively selected from the National Center for Biotechnology Information Reference Sequence (RefSeq) database (https://www.ncbi.nlm.nih.gov/refseq/) using BactinspectorMax v0.1.3 (https://gitlab.com/antunderwood/bactinspector). The selected references were the *S*. *enterica* subsp. enterica serovar Fresno strain (assembly accession: GCF_003590695.1) and the *E*. *coli* O25b:H4-ST131 strain (assembly accession: GCF_000285655.3). The sequence reads for each species were then mapped to the chromosome of the reference using BWA (v0.7.17) [38] and variants were called and filtered using bcftools (v1.9) [39] as implemented in the GHRU SNP phylogeny pipeline (https://gitlab.com/cgps/ghru/pipelines/snp_phylogeny). Variant positions were concatenated into a pseudoalignment and used to generate a maximum likelihood tree using iqtree (v1.6.8) [40]. SNP distances between the genome pairs were calculated using snp-dists v.0.8.2 (https://github.com/tseemann/snp-dists).

## Results

### *Salmonella* serotypes, sequence types (STs), virulence factors and phylogeny

**Fig 1:**
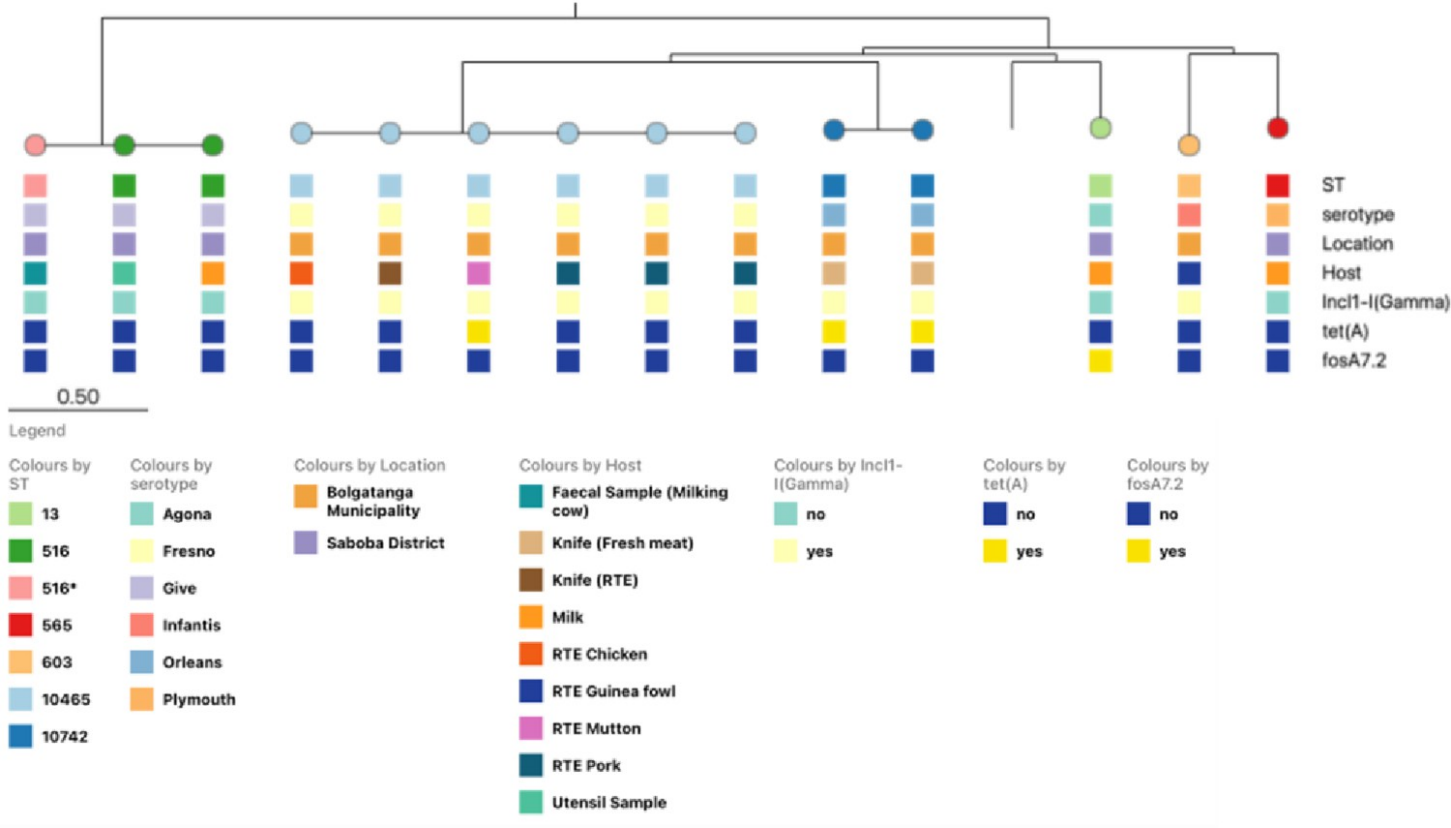
Core genome SNP-based maximum likelihood tree showing phylogenetic relationships among strains sequenced in this study. The matrix below the tree shows the sequence types, serotypes, location of isolation (Bolgatanga municipality or Saboba district) as well as the source/host sample and presence or absence of the IncI1-I(Gamma) plasmid replicon, *tetA* and *fosA* resistance genes.

We used SISTR software to predict the serovars of the 14 *Salmonella* strains characterized in this study from whole genome sequence reads, which are deposited in the European Nucleotide Archive with the study accession PRJEB58695 (S1 Table). The most common serotype was Fresno (n = 6) followed by Give (n = 3), Orleans (n = 2), Plymouth (n = 1), Agona (n = 1) and Infantis (n = 1). The *S*. Fresno and *S*. Orleans isolates were from previously unreported sequence types, now designated ST10742 and ST10465 respectively (S2 Table). As shown in Fig 1, all the isolates from ready-to-eat pork, mutton and chicken belonged to serovar Fresno. *S*. Fresno isolates were also isolated from a meat vendor’s knife, as was *S*. Orleans. The three milk isolates, which were all from Saboba, belonged to the serovars Plymouth (ST565), Give (ST516) and Agona (ST13) (Fig 1). Two more ST516 *S*. Give isolates were recovered from the faeces of milking cow and from a milking utensil.

Phylogenetic analysis of the 14 *Salmonella* isolates from this study showed that all *S*. Fresno isolates, irrespective of source, clustered together and differed by < 3 SNPs. The two *S*. Orleans isolates were identical (0 SNPs) and the three *S*. Give isolates were also identical, with the isolates originating from milk, faeces of milking cow and from a milking utensil, also in Saboba.

All *Salmonella* isolates harboured curli (*csg*) genes as well as *bcf*, *fim* and *ste* fimbrial operons and ten of them, representing all serovars except *S*. Give and Plymouth, carried long polar fimbriae (*lpf)* genes. The *S*. Infantis and *S*. Agona strains carried *ratB* and *shdA*. Type III secretion system effector genes, such as: *inv*, *org*, *prg*, *sif*, *spa*, *sse*, *ssa* and *sop* were detected in all the isolates while *avr* was present in 57.1% (8/14) of the isolates and only one isolate harboured *gogB*. Four of the isolates also encoded the cytholethal distending toxin gene, *cdtB* (S3 Table).

### Plasmid replicons and ARG profiles of *Salmonella*

The *Salmonella* isolates were largely pan-sensitive but genes conferring resistance to fosfomycin (*fosA7*.*2*) and tetracycline (*tet(A)*) were detected in one and three isolates respectively. Both *S*. Orleans isolates and one of the *S*. Fresno, from ready-to-eat mutton carried *tetA*, along with an IncI1-I(Gamma) plasmid replicon, which was also seen in six other *tetA*-negative strains. Interestingly, the IncI1-I(Gamma) plasmid replicon was detected in all isolates from Bolgatanga municipality, irrespective of serovar, and no isolate from Saboba district harboured this plasmid. The fosfomycin resistance gene was found in the *S*. Agona genome, in which no plasmid replicons were detected (Fig 1).

### *E*. *coli* serotypes, sequence types (STs), virulence factors and phylogeny

A total of 19 *E*. *coli* isolates were identified. *E*. *coli* serotyping using the ECtyper revealed that the most common serotypes among the *E*. *coli* isolates were -:H7 (n = 2), O138:H48 (n = 2), O6:H16 (n = 2), and O8/O160:H16 (n = 2). A number of these serovars and STs are associated with pathogenicity, notably O6:H16 [41], as well as O8/O160:H16 and O77/O17/O44/O106/O73:H18 (ST394; [42–44]). The strains belonging to these lineages lacked the defining virulence genes of the respective pathotypes but did contain accessory virulence genes, as shown in S4 Table.

Irrespective of whether they belonged to a lineage commonly associated with virulence, most of the isolates contained a range of adhesins and iron utilization genes. *E*. *coli* extracellular protein (ECP) export pathway (*ecp/yag*) and *ompA* and type I fimbriae-encoding operon, *fim*, encoding genes seen in most *E*. *coli* genomes, were present in 94.7% (18/19) of *E*. *coli* isolates. Fimbriae encoding gene, *f17d*, often seen in enterotoxigenic *E*. *coli* recovered from animals, was present in the two O8/O160:H16 isolates and an O-:H7 isolate.

**Fig 2:**
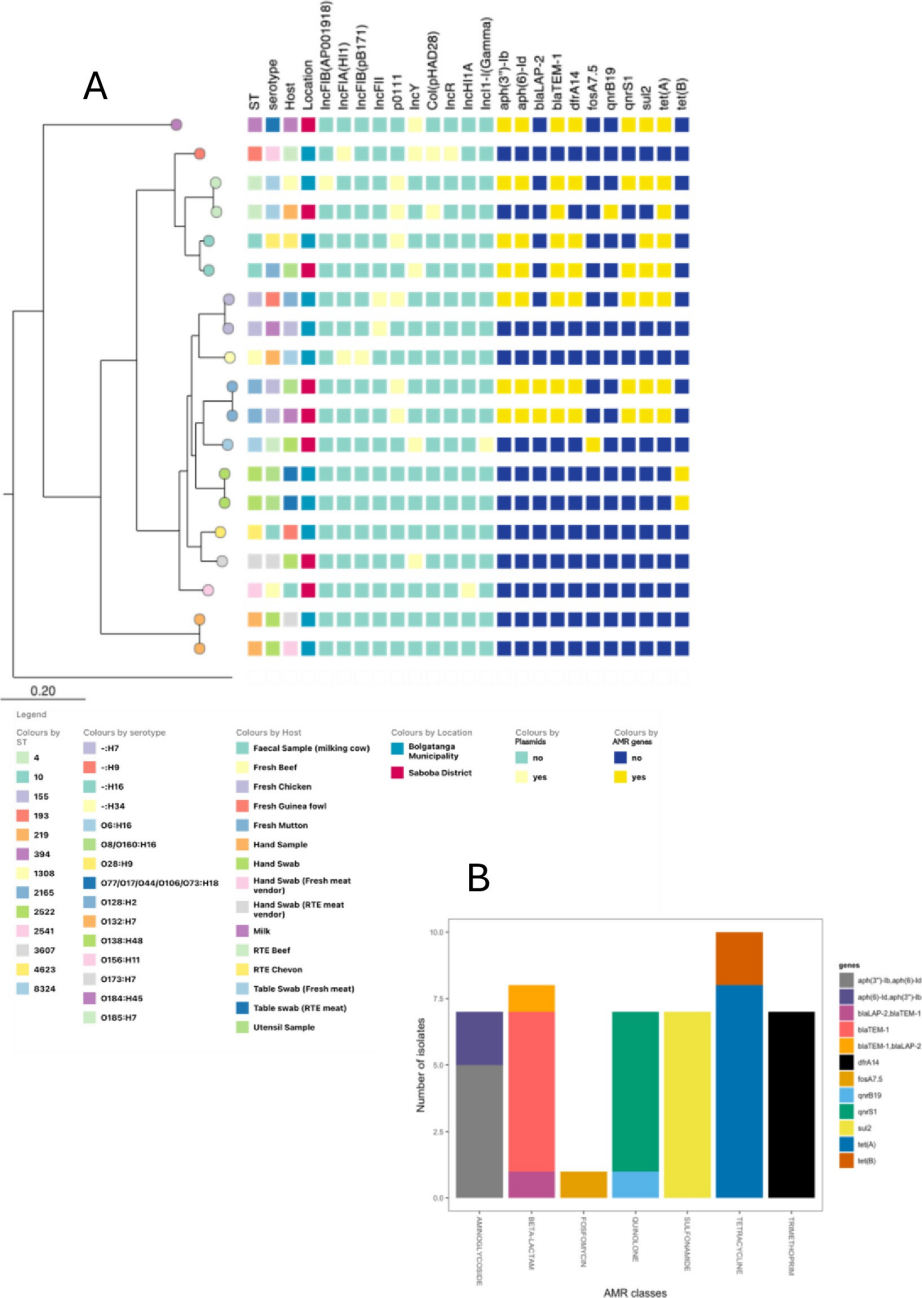
Phylogeny, plasmid replicons and resistance genes of *E*. *coli* isolates. (a). SNP-based phylogenetic tree of the *E*. *coli* isolates showing the range of sequence types, serovars, plasmid replicons and resistance genes detected (b) AMR determinants detected in *E*. *coli* and the resistance they confer.

The phylogenetic analysis of the 19 *E*. *coli* isolates from this study and a reference genome (NZ_HG941718.1) based on SNP is presented in Fig 2. The range of isolates was broader than with *Salmonella* but closely related pairs of isolates belonging to the same serovar, and ST were found in three instances. Very similar (2347 SNPs) O6:H16 isolates were recovered from different food preparation table samples in Bolgatanga. One of the two isolates from milk in Saboba belonged to ST2165 and was identical (0 SNPs) to a Saboba ST2165 utensil isolate. The two ST4 isolates from fresh beef and a cow milker’s hand differed by 2347 SNPs and are unlikely to be connected.

### Plasmid replicons and ARG profiles of *E*. *coli*

Antimicrobial resistance determinants present in the *E*. *coli* isolates include those encoding resistance to aminoglycosides (*aph(3”)-Ib*, *aph(6)-Id*, *aph(6)-Id*, *aph(3”)-Ib*), beta-lactams (*bla*_*LAP-2*_, *bla*_*TEM-1*_), fosfomycin (*fosA7*.*5*), quinolones (*qnrB19*, *qnrS1*), sulfonamide (*sul2*), tetracycline (*tet(A)*, *tet(B)*) and trimethoprim (*dfrA14*) (Fig 2). At least 3 antimicrobial resistance genes (ARGs) which confer resistance to different classes of antibiotics were present in 8 isolates. Three isolates carried one ARG each while 8 isolates had no ARGs. Six strains carried the genes *aph(3’’)-Ib*, *aph(6)-Id*, *blaTEM-1*, *dfrA14*, *sul2*, *qnrS1* and *tet(A)*. The *dfrA14-qnrS1*-*tet(A) resistance* gene combination has previously been reported from Nigeria, being part of a transposon transmitted in an IncX plasmid [45]. In this study however, IncX replicons were not detected. The most common plasmid replicon type detected among the *E*. *coli* isolates was pO111 (n = 6), originally described in an *E*. *coli* virulence plasmid and found in the aforementioned strains belonging to virulence-associated lineages. The other plasmid replicon types detected were IncY (n = 5), IncFII (n = 2), IncFIA(HI1) (n = 2), Col(pHAD28) (n = 2), IncFIB(pB171) (n = 1), IncR (n = 1), IncHI1A (n = 1) and IncI1-I(Gamma), which was common among the *Salmonella*, (n = 1) (Fig 2). All multidrug resistant *E*. *coli* strains in this study encode pO111 or IncY replicons.

## Discussion

*Salmonella* and *E*. *coli* are the main causes of bacterial foodborne illnesses in Ghana [46]. Retail meat, along with milk and their products are recognized as primary sources of foodborne Salmonellosis [12] and *E*. *coli* infection [7]. Post-cooking handling practices, exposure during the points of sale, and suboptimal meat storage conditions collectively contribute to an increased presence of both pathogenic and spoilage bacteria in ready-to-eat (RTE) meat [47]. Within Ghana, food safety has been inadequately studied in the northern region [10]. In this study, we characterized the genomes of 14 *Salmonella* and 19 *E*. *coli* previously isolated from Saboba district and Bolgatanga Municipality in northern Ghana.

The *Salmonella* serovars (Fresno, Plymouth, Infantis, Give and Orleans) identified in this study are yet to be reported in Ghana, but Guinee et al. (1961) [48] isolated *S*. Agona from cattle in Ghana and *S*. Give (but ST524, different from ST516 in this study) has been reported from beef in Nigeria [49]. Isolation of *S*. Infantis from retail poultry meat has also been reported in Ecuador [50], Belgium [51] and Italy [52] and all the serovars detected in this study have been implicated in human infections. The identification of *Salmonella* serovars not previously documented in the country in meat and milk products highlights the need for heightened surveillance and preventive measures to curb the spread of foodborne pathogens and reduce the risk of associated illnesses.

Unlike *Salmonella*, not all *E*. *coli* are potential pathogens. However, *E*. *coli* serve as markers for faecal contamination and therefore the potential that other pathogens are present. The predominant *E*. *coli* STs (ST4, ST10, ST219, ST2522) detected in our study have been previously isolated from food animals and have been associated with pathogenicity [53–55].

While none of the *E*. *coli* isolates carried genes encoding ETEC heat-sensitive or heat-labile enterotoxins, f17d fimbrial genes present in four of the *E*. *coli* genomes encode ETEC colonization factors commonly associated with colonization of cattle and other ruminant isolates [56]. *E*. *coli*, F17 fimbriae are associated with pathogenic *E*. *coli* recovered from diarrhoea and septicaemia outbreaks in calves, lambs, and humans, including from outbreaks.

Additionally, two of the ST4 *E*. *coli* isolates from this study not harbouring f17d fimbrial genes belong to the serovar O6:H16, one of the most widely disseminated lineages of human enterotoxigenic *E*. *coli* (ETEC). O6:H16 ETEC cause outbreaks, often associated with food and/or inadequate handwashing [57–59]. ETEC, by definition, produce plasmid-encoded heat-labile and/or heat stable toxins not present in the genomes of the isolates from this study.

However, the serovars O8/O160:H16 and O77/O17/O44/O106/O73:H18 belong to a previously described enteroaggregative *E*. *coli* (EAEC) lineage [42, 44]. Isolates from this study belonging to the serovars O8/O160:H16 and O77/O17/O44/O106/O73:H18 possessed no EAEC accessory genes. These strains are from virulent lineages but lack key virulence genes that are plasmid-encoded, which could mean that these plasmids were lost in the food chain or during isolation but could be reacquired. Nevertheless, the presence of these strains in food could increase the risk of foodborne illness.

While strains belonging to virulence-associated lineages lacked key plasmid-encoded virulence plasmids, several plasmid replicons were detected in the isolate genomes. According to McMillan et al. (2019) [60], plasmid replicons ColE, IncI1, IncF, and IncX were commonly detected in *Salmonella* from food animals in the US. In this study, the IncI1 replicon was predominant, with nine of the thirteen *Salmonella* strains harbouring IncI1 plasmid replicon, of which three harboured the *tetA* gene. This is likely to be an instance of a successful mobile element with extraordinary local reach, a few of which have been reported from West Africa, including Ghana, in the past [45, 61–63]. The IncI1-I(Gamma) plasmid replicon observed among *Salmonella* isolates was detected in all *Salmonella* isolates from Bolgatanga municipality—three different serovars—and none of the Saboba district isolates. However, one *E*. *coli* isolate (from a recently reported ST, ST8274) from Saboba did have this replicon. As it is an IncI1 plasmid replicon, its plasmid should be better characterized and, it should remain under surveillance because numerous articles have reported association of the IncI1 plasmid replicon with multiple ARGs, such as *tetB*, *tetAR*, *bla*_*CMY*-2_, *bla*_*TEM*-1_, *aac3VIa*, *aphA*, *aadA* and *sul1* [60], *bla*_*CTXM*-1_ [64], *strA*, *strB*, *cmlA*, *floR*, *bla*_*SHV-12*_, *bla*_*OXA-2*_ and *FosA3* [65] in IncI1 plasmids in *Salmonella*. As our own sequence was generated by short read only, the first step would be to generate long read sequence that could fully assemble the plasmid and make it possible to identify genetic factors supporting its success.

Among the *E*. *coli* isolates, the plasmid replicons pO111 was the most common replicon. A previous study by Balbuena-Alonso et al. (2022) [66], revealed that pO111 is usually associated with extended spectrum beta lactamases gene and is very common in food and clinical isolates. In this study, all isolates carrying pO111 harbour at least one beta-lactamase gene. Likewise, all the pO111 plasmid bearing isolates in this study carried ARGs that confer resistance to at least 4 classes of antibiotics. Altogether, these data demonstrate a concerning reservoir of resistance genes in these foodborne bacteria.

## Conclusion

This study has characterized the genomes of *Salmonella* and *E*. *coli* in milk, meat and their associated utensils. The diverse serovars and virulence genes detected in *Salmonella* strains indicate potential pathogenicity. Although not all *E*. *coli* strains are pathogenic, their presence serves as an indicator of faecal contamination, suggesting the potential presence of other harmful pathogens. The presence of EAEC strains in food is concerning as EAEC is a well-known cause of diarrhoeal diseases, particularly in children and immunocompromised individuals, making its presence in food a serious concern. While antimicrobial resistance was not common among *Salmonella* strains, most of the *E*. *coli* strain had at least one resistance gene, and almost half were multidrug resistant and carried mobile elements. Moreover, there have been recent reports of resistant *Salmonella* and E. *coli* from meat and milk elsewhere in West Africa [49].

A recent scoping review reported weak enforcement of food safety regulations, as well as a lack of infrastructure, knowledge and skills to implement these regulations [10, 11, 46]. Food contaminated with and *Salmonella* and *E*. *coli* can serve as a vehicle for their transmission, posing a significant public health risk. We recommend that food safety regulations be strengthened in northern Ghana and, by extension, West Africa. It is also important to increase awareness among consumers so that food is handled in such a way to prevent pathogen transmission. There is an additional need for continuous surveillance and preventive measures to stop the spread of foodborne pathogens and reduce the risk of associated illnesses in Ghana.

## Supporting information

S1 Table Accession numbers for genomes generated in this study. (XLSX)

S2 Table Novel *Salmonella* allelic profile and assigned ST. (XLSX)

S3 Table *Salmonella* strain metadata including serotype, ST, plasmid replicon, AMR and virulence profile. (XLSX)

S4 Table *E*. *coli* strain metadata including serotype, ST, plasmid replicon, AMR and virulence profile. (XLSX)

## References

[1] Grace D, Wu F, Havelaar AH. MILK Symposium review: Foodborne diseases from milk and milk products in developing countries-Review of causes and health and economic implications. J Dairy Sci. 2020;103(11):9715–29. doi: 10.3168/jds.2020-18323 33076183

[2] Abraham S, O’Dea M, Sahibzada S, Hewson K, Pavic A, Veltman T, et al. Escherichia coli and Salmonella spp. isolated from Australian meat chickens remain susceptible to critically important antimicrobial agents. PLoS One. 2019;14(10):e0224281. doi: 10.1371/journal.pone.0224281 31644602 PMC6808415

[3] Elzhraa F, Al-Ashmawy M, El-Sherbini M, Abdelkhalek A. Critical occurrence of verotoxgenic E.coli and non-typhoidal salmonella in some heat treated dairy products. Ital J Food Saf. 2021;10(2):9318. doi: 10.4081/ijfs.2021.9318 34268144 PMC8256307

[4] Gutema FD, Abdi RD, Agga GE, Firew S, Rasschaert G, Mattheus W, et al. Assessment of beef carcass contamination with Salmonella and E. coli O 157 in slaughterhouses in Bishoftu, Ethiopia. International Journal of Food Contamination. 2021;8:1–9.

[5] Lay KK, Jeamsripong S, Sunn KP, Angkititrakul S, Prathan R, Srisanga S, et al. Colistin Resistance and ESBL Production in Salmonella and Escherichia coli from Pigs and Pork in the Thailand, Cambodia, Lao PDR, and Myanmar Border Area. Antibiotics (Basel). 2021;10(6). doi: 10.3390/antibiotics10060657 34072965 PMC8226727

[6] Parry-Hanson Kunadu A, Holmes M, Miller EL, Grant AJ. Microbiological quality and antimicrobial resistance characterization of Salmonella spp. in fresh milk value chains in Ghana. Int J Food Microbiol. 2018;277:41–9. doi: 10.1016/j.ijfoodmicro.2018.04.025 29680695

[7] Adzitey F, Asante J, Kumalo HM, Khan RB, Somboro AM, Amoako DG. Genomic Investigation into the Virulome, Pathogenicity, Stress Response Factors, Clonal Lineages, and Phylogenetic Relationship of Escherichia coli Strains Isolated from Meat Sources in Ghana. Genes (Basel). 2020;11(12). doi: 10.3390/genes11121504 33327465 PMC7764966

[8] Aduah M, Adzitey F, Amoako DG, Abia ALK, Ekli R, Teye GA, et al. Not All Street Food Is Bad: Low Prevalence of Antibiotic-Resistant Salmonella enterica in Ready-to-Eat (RTE) Meats in Ghana Is Associated with Good Vendors’ Knowledge of Meat Safety. Foods. 2021;10(5). doi: 10.3390/foods10051011 34066440 PMC8148193

[9] Tay MYF, Adzitey F, Sultan SA, Tati JM, Seow KLG, Schlundt J. Whole-Genome Sequencing of Nontyphoidal Salmonella enterica Isolates Obtained from Various Meat Types in Ghana. Microbiol Resour Announc. 2019;8(15). doi: 10.1128/MRA.00033-19 30975795 PMC6460018

[10] Botha NN, Ansah EW, Segbedzi CE, Darkwa S. Public health concerns for food contamination in Ghana: A scoping review. PLoS One. 2023;18(8):e0288685. doi: 10.1371/journal.pone.0288685 37561804 PMC10414628

[11] Asati DA, Abdulai PM, Boateng KS, Appau AAA, Ofori LA, Agyekum TP. Food safety knowledge and practices among raw meat handlers and the microbial content of raw meat sold at Kumasi Abattoir Butchery Shops in Kumasi, Ghana. BMC Public Health. 2024;24(1):975. doi: 10.1186/s12889-024-18514-w 38584288 PMC11000319

[12] Adzitey F, Assoah-Peprah P, Teye GA. Whole-genome sequencing of Escherichia coli isolated from contaminated meat samples collected from the Northern Region of Ghana reveals the presence of multiple antimicrobial resistance genes. J Glob Antimicrob Resist. 2019;18:179–82. doi: 10.1016/j.jgar.2019.03.014 30926467

[13] Adzitey F, Teye GA, Amoako DG. Prevalence, phylogenomic insights, and phenotypic characterization of Salmonella enterica isolated from meats in the Tamale metropolis of Ghana. Food Sci Nutr. 2020;8(7):3647–55. doi: 10.1002/fsn3.1647 32724627 PMC7382109

[14] Falgenhauer L, Imirzalioglu C, Oppong K, Akenten CW, Hogan B, Krumkamp R, et al. Detection and Characterization of ESBL-Producing Escherichia coli From Humans and Poultry in Ghana. Front Microbiol. 2018;9:3358. doi: 10.3389/fmicb.2018.03358 30697208 PMC6340976

[15] Adzitey F, Yussif S, Ayamga R, Zuberu S, Addy F, Adu-Bonsu G, et al. Antimicrobial Susceptibility and Molecular Characterization of Escherichia coli Recovered from Milk and Related Samples. Microorganisms. 2022;10(7). doi: 10.3390/microorganisms10071335 35889054 PMC9320388

[16] Moawad AA, Hotzel H, Awad O, Tomaso H, Neubauer H, Hafez HM, et al. Occurrence of Salmonella enterica and Escherichia coli in raw chicken and beef meat in northern Egypt and dissemination of their antibiotic resistance markers. Gut Pathog. 2017;9:57. doi: 10.1186/s13099-017-0206-9 29075329 PMC5648511

[17] Geletu US, Usmael MA, Ibrahim AM. Isolation, Identification, and Susceptibility Profile of E. coli, Salmonella, and S. aureus in Dairy Farm and Their Public Health Implication in Central Ethiopia. Vet Med Int. 2022;2022:1887977. doi: 10.1155/2022/1887977 35198138 PMC8860541

[18] Worku W, Desta M, Menjetta T. High prevalence and antimicrobial susceptibility pattern of salmonella species and extended-spectrum beta-lactamase producing Escherichia coli from raw cattle meat at butcher houses in Hawassa city, Sidama regional state, Ethiopia. PLoS One. 2022;17(1):e0262308. 35030183 10.1371/journal.pone.0262308 PMC8759633

[19] Lyu N, Feng Y, Pan Y, Huang H, Liu Y, Xue C, et al. Genomic Characterization of Salmonella enterica Isolates From Retail Meat in Beijing, China. Front Microbiol. 2021;12:636332. doi: 10.3389/fmicb.2021.636332 33897640 PMC8058101

[20] Zhan Z, Xu X, Gu Z, Meng J, Wufuer X, Wang M, et al. Molecular epidemiology and antimicrobial resistance of invasive non-typhoidal Salmonella in China, 2007–2016. Infect Drug Resist. 2019;12:2885–97. doi: 10.2147/IDR.S210961 31571942 PMC6750164

[21] Jaja IF, Bhembe NL, Green E, Oguttu J, Muchenje V. Molecular characterisation of antibiotic-resistant Salmonella enterica isolates recovered from meat in South Africa. Acta Trop. 2019;190:129–36. doi: 10.1016/j.actatropica.2018.11.003 30408462

[22] Rantsiou K, Kathariou S, Winkler A, Skandamis P, Saint-Cyr MJ, Rouzeau-Szynalski K, et al. Next generation microbiological risk assessment: opportunities of whole genome sequencing (WGS) for foodborne pathogen surveillance, source tracking and risk assessment. Int J Food Microbiol. 2018;287:3–9. doi: 10.1016/j.ijfoodmicro.2017.11.007 29246458

[23] Hilt EE, Ferrieri P. Next Generation and Other Sequencing Technologies in Diagnostic Microbiology and Infectious Diseases. Genes (Basel). 2022;13(9). doi: 10.3390/genes13091566 36140733 PMC9498426

[24] Nadon C, Van Walle I, Gerner-Smidt P, Campos J, Chinen I, Concepcion-Acevedo J, et al. PulseNet International: Vision for the implementation of whole genome sequencing (WGS) for global food-borne disease surveillance. Euro Surveill. 2017;22(23). doi: 10.2807/1560-7917.ES.2017.22.23.30544 28662764 PMC5479977

[25] Saba CK, Escudero JA, Herrera-Leon S, Porrero MC, Suarez M, Dominguez L, et al. First identification of Salmonella Urbana and Salmonella Ouakam in humans in Africa. J Infect Dev Ctries. 2013;7(10):691–5. doi: 10.3855/jidc.3548 24129620

[26] Abass A, Adzitey F, Huda N. Escherichia coli of Ready-to-Eat (RTE) Meats Origin Showed Resistance to Antibiotics Used by Farmers. Antibiotics (Basel). 2020;9(12). doi: 10.3390/antibiotics9120869 33291648 PMC7761968

[27] Tibile AB. Incidence of Salmonella enterica and total bacterial count obtained from cow milk and its related samples from Saboba district: One health approach [A dissertation]: University for Development Studies, Ghana; 2022.

[28] Rahn K, De Grandis SA, Clarke RC, McEwen SA, Galan JE, Ginocchio C, et al. Amplification of an invA gene sequence of Salmonella typhimurium by polymerase chain reaction as a specific method of detection of Salmonella. Mol Cell Probes. 1992;6(4):271–9. doi: 10.1016/0890-8508(92)90002-f 1528198

[29] Prjibelski A, Antipov D, Meleshko D, Lapidus A, Korobeynikov A. Using SPAdes De Novo Assembler. Curr Protoc Bioinformatics. 2020;70(1):e102. doi: 10.1002/cpbi.102 32559359

[30] Zhou Z, Alikhan NF, Mohamed K, Fan Y, Agama Study G, Achtman M. The EnteroBase user’s guide, with case studies on Salmonella transmissions, Yersinia pestis phylogeny, and Escherichia core genomic diversity. Genome Res. 2020;30(1):138–52. doi: 10.1101/gr.251678.119 31809257 PMC6961584

[31] Ikhimiukor OO, Oaikhena AO, Afolayan AO, Fadeyi A, Kehinde A, Ogunleye VO, et al. Genomic characterization of invasive typhoidal and non-typhoidal Salmonella in southwestern Nigeria. PLoS Negl Trop Dis. 2022;16(8):e0010716. doi: 10.1371/journal.pntd.0010716 36026470 PMC9455843

[32] Akinlabi OC, Nwoko EQ, Dada RA, Ekpo S, Omotuyi A, Nwimo CC, et al. Epidemiology and Risk Factors for Diarrheagenic Escherichia coli Carriage among Children in Northern Ibadan, Nigeria. Am J Trop Med Hyg. 2023;109(6):1223–32. doi: 10.4269/ajtmh.22-0618 37903436 PMC10793065

[33] Hunt M, Mather AE, Sanchez-Buso L, Page AJ, Parkhill J, Keane JA, et al. ARIBA: rapid antimicrobial resistance genotyping directly from sequencing reads. Microb Genom. 2017;3(10):e000131. doi: 10.1099/mgen.0.000131 29177089 PMC5695208

[34] Yoshida CE, Kruczkiewicz P, Laing CR, Lingohr EJ, Gannon VP, Nash JH, et al. The Salmonella In Silico Typing Resource (SISTR): An Open Web-Accessible Tool for Rapidly Typing and Subtyping Draft Salmonella Genome Assemblies. PLoS One. 2016;11(1):e0147101. doi: 10.1371/journal.pone.0147101 26800248 PMC4723315

[35] Bessonov K, Laing C, Robertson J, Yong I, Ziebell K, Gannon VPJ, et al. ECTyper: in silico Escherichia coli serotype and species prediction from raw and assembled whole-genome sequence data. Microb Genom. 2021;7(12). doi: 10.1099/mgen.0.000728 34860150 PMC8767331

[36] Carattoli A, Zankari E, Garcia-Fernandez A, Voldby Larsen M, Lund O, Villa L, et al. In silico detection and typing of plasmids using PlasmidFinder and plasmid multilocus sequence typing. Antimicrob Agents Chemother. 2014;58(7):3895–903. doi: 10.1128/AAC.02412-14 24777092 PMC4068535

[37] Feldgarden M, Brover V, Gonzalez-Escalona N, Frye JG, Haendiges J, Haft DH, et al. AMRFinderPlus and the Reference Gene Catalog facilitate examination of the genomic links among antimicrobial resistance, stress response, and virulence. Sci Rep. 2021;11(1):12728. doi: 10.1038/s41598-021-91456-0 34135355 PMC8208984

[38] Li H, Durbin R. Fast and accurate short read alignment with Burrows-Wheeler transform. Bioinformatics. 2009;25(14):1754–60. doi: 10.1093/bioinformatics/btp324 19451168 PMC2705234

[39] Danecek P, Bonfield JK, Liddle J, Marshall J, Ohan V, Pollard MO, et al. Twelve years of SAMtools and BCFtools. Gigascience. 2021;10(2). doi: 10.1093/gigascience/giab008 33590861 PMC7931819

[40] Nguyen LT, Schmidt HA, von Haeseler A, Minh BQ. IQ-TREE: a fast and effective stochastic algorithm for estimating maximum-likelihood phylogenies. Mol Biol Evol. 2015;32(1):268–74. doi: 10.1093/molbev/msu300 25371430 PMC4271533

[41] Scotland SM, Gross RJ, Rowe B. Serotype-related enterotoxigenicity in Escherichia coli O6.H16 and O148.H28. J Hyg (Lond). 1977;79(3):395–403. doi: 10.1017/s0022172400053249 336791 PMC2129966

[42] Okeke IN, Wallace-Gadsden F, Simons HR, Matthews N, Labar AS, Hwang J, et al. Multi-locus sequence typing of enteroaggregative Escherichia coli isolates from Nigerian children uncovers multiple lineages. PLoS One. 2010;5(11):e14093. doi: 10.1371/journal.pone.0014093 21124856 PMC2990770

[43] Chattaway MA, Day M, Mtwale J, White E, Rogers J, Day M, et al. Clonality, virulence and antimicrobial resistance of enteroaggregative Escherichia coli from Mirzapur, Bangladesh. J Med Microbiol. 2017;66(10):1429–35. doi: 10.1099/jmm.0.000594 28945190 PMC5845566

[44] Chen Y, Chen X, Zheng S, Yu F, Kong H, Yang Q, et al. Serotypes, genotypes and antimicrobial resistance patterns of human diarrhoeagenic Escherichia coli isolates circulating in southeastern China. Clin Microbiol Infect. 2014;20(1):52–8. doi: 10.1111/1469-0691.12188 23521436

[45] Sumrall ET, Gallo EB, Aboderin AO, Lamikanra A, Okeke IN. Dissemination of the transmissible quinolone-resistance gene qnrS1 by IncX plasmids in Nigeria. PLoS One. 2014;9(10):e110279. doi: 10.1371/journal.pone.0110279 25340787 PMC4207749

[46] Christiana Cudjoe D, Balali GI, Titus OO, Osafo R, Taufiq M. Food Safety in Sub-Sahara Africa, An insight into Ghana and Nigeria. Environ Health Insights. 2022;16:11786302221142484. doi: 10.1177/11786302221142484 36530486 PMC9755555

[47] Henriques A, Fraqueza M. Listeria monocytogenes and ready-to-eat meat-based food products: Incidence and control. Listeria monocytogenes: incidence, growth behavior and control. 2015:71–103.

[48] Guinee PA, Kampelmacher EH, Willems HM. Six new Salmonella types, isolated in Ghana (S. volta, S. agona, S. wa, S. techimani, S. mampong and S. tafo). Antonie Van Leeuwenhoek. 1961;27:469–72. doi: 10.1007/BF02538474 13902866

[49] Aworh MK, Nilsson P, Egyir B, Owusu FA, Hendriksen RS. Rare serovars of non-typhoidal Salmonella enterica isolated from humans, beef cattle and abattoir environments in Nigeria. PLoS One. 2024;19(1):e0296971. doi: 10.1371/journal.pone.0296971 38252613 PMC10802957

[50] Mejia L, Vela G, Zapata S. High Occurrence of Multiresistant Salmonella Infantis in Retail Meat in Ecuador. Foodborne Pathog Dis. 2021;18(1):41–8. doi: 10.1089/fpd.2020.2808 32808817

[51] Zeng H, De Reu K, Gabriel S, Mattheus W, De Zutter L, Rasschaert G. Salmonella prevalence and persistence in industrialized poultry slaughterhouses. Poult Sci. 2021;100(4):100991. doi: 10.1016/j.psj.2021.01.014 33610890 PMC7905466

[52] Casagrande Proietti P, Musa L, Stefanetti V, Orsini M, Toppi V, Branciari R, et al. mcr-1-Mediated Colistin Resistance and Genomic Characterization of Antimicrobial Resistance in ESBL-Producing Salmonella Infantis Strains from a Broiler Meat Production Chain in Italy. Antibiotics (Basel). 2022;11(6). doi: 10.3390/antibiotics11060728 35740135 PMC9220226

[53] Araujo S, I ATS, Tacao M, Patinha C, Alves A, Henriques I. Characterization of antibiotic resistant and pathogenic Escherichia coli in irrigation water and vegetables in household farms. Int J Food Microbiol. 2017;257:192–200. doi: 10.1016/j.ijfoodmicro.2017.06.020 28668729

[54] Silva A, Silva V, Pereira JE, Maltez L, Igrejas G, Valentao P, et al. Antimicrobial Resistance and Clonal Lineages of Escherichia coli from Food-Producing Animals. Antibiotics (Basel). 2023;12(6). doi: 10.3390/antibiotics12061061 37370379 PMC10295564

[55] Zhao X, Miao Y, Adam FEA, Zhao H, Zhou Z, Su M, et al. ESBLs-producing Escherichia coli from sheep-origin: Genome-wide virulence genes identification and in vivo virulence assessment in mice and Galleria mellonella. Transbound Emerg Dis. 2022;69(6):3606–17. doi: 10.1111/tbed.14729 36222239

[56] Bihannic M, Ghanbarpour R, Auvray F, Cavalie L, Chatre P, Boury M, et al. Identification and detection of three new F17 fimbrial variants in Escherichia coli strains isolated from cattle. Vet Res. 2014;45(1):76. doi: 10.1186/s13567-014-0076-9 25106491 PMC4267768

[57] Pacheco AB, Soares KC, de Almeida DF, Viboud GI, Binsztein N, Ferreira LC. Clonal nature of enterotoxigenic Escherichia coli serotype O6: H16 revealed by randomly amplified polymorphic DNA analysis. Journal of clinical microbiology. 1998;36(7):2099–102. doi: 10.1128/JCM.36.7.2099-2102.1998 9650973 PMC104989

[58] Beatty ME, Adcock PM, Smith SW, Quinlan K, Kamimoto LA, Rowe SY, et al. Epidemic diarrhea due to enterotoxigenic Escherichia coli. Clin Infect Dis. 2006;42(3):329–34. doi: 10.1086/499246 16392076

[59] Willshaw GA, Cheasty T, Rowe B, Smith HR, Faithfull-Davies DN, Brooks TG. Isolation of enterotoxigenic Escherichia coli from British troops in Saudi Arabia. Epidemiol Infect. 1995;115(3):455–63. doi: 10.1017/s0950268800058611 8557077 PMC2271586

[60] McMillan EA, Gupta SK, Williams LE, Jove T, Hiott LM, Woodley TA, et al. Antimicrobial Resistance Genes, Cassettes, and Plasmids Present in Salmonella enterica Associated With United States Food Animals. Front Microbiol. 2019;10:832. doi: 10.3389/fmicb.2019.00832 31057528 PMC6479191

[61] Dekker D, Eibach D, Boahen KG, Akenten CW, Pfeifer Y, Zautner AE, et al. Fluoroquinolone-Resistant Salmonella enterica, Campylobacter spp., and Arcobacter butzleri from Local and Imported Poultry Meat in Kumasi, Ghana. Foodborne Pathog Dis. 2019;16(5):352–8. doi: 10.1089/fpd.2018.2562 30907631 PMC6529854

[62] Labar AS, Millman JS, Ruebush E, Opintan JA, Bishar RA, Aboderin AO, et al. Regional dissemination of a trimethoprim-resistance gene cassette via a successful transposable element. PLoS One. 2012;7(5):e38142. doi: 10.1371/journal.pone.0038142 22666464 PMC3364232

[63] Monarrez R, Braun M, Coburn-Flynn O, Botelho J, Odetoyin BW, Otero-Vera JI, et al. A large self-transmissible resistance plasmid from Nigeria contains genes that ameliorate a carrying cost. Sci Rep. 2019;9(1):19624. doi: 10.1038/s41598-019-56064-z 31873110 PMC6927977

[64] Abraham S, Kirkwood RN, Laird T, Saputra S, Mitchell T, Singh M, et al. Dissemination and persistence of extended-spectrum cephalosporin-resistance encoding IncI1-bla(CTXM-1) plasmid among Escherichia coli in pigs. ISME J. 2018;12(10):2352–62. doi: 10.1038/s41396-018-0200-3 29899511 PMC6155088

[65] Foley SL, Kaldhone PR, Ricke SC, Han J. Incompatibility Group I1 (IncI1) Plasmids: Their Genetics, Biology, and Public Health Relevance. Microbiol Mol Biol Rev. 2021;85(2). doi: 10.1128/MMBR.00031-20 33910982 PMC8139525

[66] Balbuena-Alonso MG, Cortes-Cortes G, Kim JW, Lozano-Zarain P, Camps M, Del Carmen Rocha-Gracia R. Genomic analysis of plasmid content in food isolates of E. coli strongly supports its role as a reservoir for the horizontal transfer of virulence and antibiotic resistance genes. Plasmid. 2022;123–124:102650. doi: 10.1016/j.plasmid.2022.102650 36130651 PMC10896638

