# Supplementary material for: Genomic characterization of foodborne Salmonella enterica and Escherichia coli isolates from Saboba district and Bolgatanga Municipality Ghana
Source: PLoS One. 2025 Feb 7;20(2):e0315583. doi: 10.1371/journal.pone.0315583 (PMC11805442; doi:10.1371/journal.pone.0315583)
Supplement: Attachment — Submitted filename: PONE-D-24-40234.pdf [file pone.0315583.s006.pdf]

PLOS ONE

# Genomic characterization of foodborne Salmonella enterica and Escherichia coli isolates from Saboba district and Bolgatanga Municipality Ghana

--Manuscript Draft--

| | |
|---|---|
| **Manuscript Number:** | PONE-D-24-40234 |
| **Article Type:** | Research Article |
| **Full Title:** | Genomic characterization of foodborne Salmonella enterica and Escherichia coli isolates from Saboba district and Bolgatanga Municipality Ghana |
| **Short Title:** | Foodborne Salmonella and E. coli from Ghana |
| **Corresponding Author:** | Iruka N Okeke<br>University of Ibadan<br>NIGERIA |
| **Keywords:** | Salmonella enterica; Escherichia coli; Ghana; Milk; Meat; Food Safety; Antimicrobial Resistance; antibiotic resistance; Plasmids |
| **Abstract:** | Salmonella and Escherichia coli are well-known bacteria commonly associated with foodborne illnesses in humans and animals. Genomic characterization of these pathogens provides valuable insights into their evolution, virulence factors and resistance determinants. This study characterized Salmonella (n = 14) and E. coli (n = 19) from milk, meat and its associated utensils in Ghana using whole-genome sequencing. Most of the Salmonella serovars (Fresno, Plymouth, Infantis, Give and Orleans) identified in this study are yet to be reported in Ghana. The Salmonella enterica isolates were largely pan-sensitive but genes conferring resistance to fosfomycin (fosA7.2) and tetracycline (tet(A)) were detected in one and three isolates respectively. While 7of the Salmonella isolates carry the IncI1-I(Gamma) plasmid replicon. While antimicrobial resistance was not common among Salmonella strains, most (11/19) of the E. coli strains had at least one resistance gene and almost half (8/19) were multidrug resistance and carry mobile elements. Three of the 19 E. coli strains belong to serovars commonly associated with enteroaggregative E. coli (EAEC) lineage. While strains belonging to virulence-associated lineages lacked key plasmid-encoded virulence plasmids, several plasmid replicons were detected in most of the E. coli (14/19) strains. Food contaminated with these pathogens can serve as a vehicle for transmission, posing a significant public health risk and necessitating stringent food safety and hygiene practices to prevent outbreaks. Hence, there is need for continuous surveillance and preventive measures to stop the spread of foodborne pathogens and reduce the risk of associated illnesses in Ghana. |
| **Order of Authors:** | Gabriel T Sunmonu |
| | Frederick Adzitey |
| | Erkison Ewomazino Odih |
| | Boniface A Tibile |
| | Rejoice Ekli |
| | Martin Aduah |
| | Anderson O Oaikhena |
| | Olabisi C Akinlabi |
| | Akebe Luther King Abia |
| | Daniel Gyamfi Amoako |
| | Iruka N Okeke |
| **Additional Information:** | |
| **Question** | **Response** |
| **Financial Disclosure** | Yes |

Powered by Editorial Manager® and ProduXion Manager® from Aries Systems Corporation

| | |
|---|---|
| Enter a financial disclosure statement that describes the sources of funding for the work included in this submission. Review the submission guidelines for detailed requirements. View published research articles from *PLOS ONE* for specific examples.<br><br>This statement is required for submission and **will appear in the published article** if the submission is accepted. Please make sure it is accurate.<br><br>**Funded studies**<br>Enter a statement with the following details:<br>• Initials of the authors who received each award<br>• Grant numbers awarded to each author<br>• The full name of each funder<br>• URL of each funder website<br>• Did the sponsors or funders play any role in the study design, data collection and analysis, decision to publish, or preparation of the manuscript?<br><br>Did you receive funding for this work? | |
| Please add funding details.<br>as follow-up to "**Financial Disclosure**<br><br>Enter a financial disclosure statement that describes the sources of funding for the work included in this submission. Review the submission guidelines for detailed requirements. View published research articles from *PLOS ONE* for specific examples.<br><br>This statement is required for submission and **will appear in the published article** if the submission is accepted. Please make sure it is accurate. | Whole genome sequencing and analysis for this project was supported through SEQAFRICA award to INO. The SEQAFRICA project is funded by the Department of Health and Social Care's Fleming Fund using UK aid. The views expressed in this publication are those of the authors and not necessarily those of the UK Department of Health and Social Care or its Management Agent, Mott MacDonald. INO is a Calestous Juma Fellow supported by the Bill and Melinda Gates Foundation. |

Powered by Editorial Manager® and ProduXion Manager® from Aries Systems Corporation

| | |
|---|---|
| **Funded studies**<br>Enter a statement with the following details:<br>• Initials of the authors who received each award<br>• Grant numbers awarded to each author<br>• The full name of each funder<br>• URL of each funder website<br>• Did the sponsors or funders play any role in the study design, data collection and analysis, decision to publish, or preparation of the manuscript?<br><br>Did you receive funding for this work?" | |
| Please select the country of your main research funder (please select carefully as in some cases this is used in fee calculation).<br>as follow-up to "**Financial Disclosure**<br><br>Enter a financial disclosure statement that describes the sources of funding for the work included in this submission. Review the submission guidelines for detailed requirements. View published research articles from *PLOS ONE* for specific examples.<br><br>This statement is required for submission and **will appear in the published article** if the submission is accepted. Please make sure it is accurate.<br><br>**Funded studies**<br>Enter a statement with the following details:<br>• Initials of the authors who received each award<br>• Grant numbers awarded to each author<br>• The full name of each funder<br>• URL of each funder website<br>• Did the sponsors or funders play any role in the study design, data collection and analysis, decision to publish, or preparation of the manuscript?<br><br>Did you receive funding for this work?" | UNITED KINGDOM - GB |
| **Competing Interests** | The authors have declared that no competing interests exist. |

| | |
|---|---|
| Use the instructions below to enter a competing interest statement for this submission. On behalf of all authors, disclose any competing interests that could be perceived to bias this work—acknowledging all financial support and any other relevant financial or non-financial competing interests.<br><br>This statement is required for submission and **will appear in the published article** if the submission is accepted. Please make sure it is accurate and that any funding sources listed in your Funding Information later in the submission form are also declared in your Financial Disclosure statement.<br><br>View published research articles from *PLOS ONE* for specific examples.<br><br>**NO authors have competing interests**<br><br>Enter: *The authors have declared that no competing interests exist.*<br><br>**Authors with competing interests**<br><br>Enter competing interest details beginning with this statement:<br><br>*I have read the journal's policy and the authors of this manuscript have the following competing interests: [insert competing interests here]*<br><br>* typeset | |
| **Ethics Statement**<br><br>Enter an ethics statement for this submission. This statement is required if the study involved:<br><br>• Human participants<br>• Human specimens or tissue<br>• Vertebrate animals or cephalopods<br>• Vertebrate embryos or tissues | N/A |

| | |
|---|---|
| • Field research<br><br>Write "N/A" if the submission does not require an ethics statement.<br><br>General guidance is provided below. Consult the submission guidelines for detailed instructions. **Make sure that all information entered here is included in the Methods section of the manuscript.** | |

| | |
|---|---|
| **Format for specific study types**<br><br>**Human Subject Research (involving human participants and/or tissue)**<br>• Give the name of the institutional review board or ethics committee that approved the study<br>• Include the approval number and/or a statement indicating approval of this research<br>• Indicate the form of consent obtained (written/oral) or the reason that consent was not obtained (e.g. the data were analyzed anonymously)<br><br>**Animal Research (involving vertebrate animals, embryos or tissues)**<br>• Provide the name of the Institutional Animal Care and Use Committee (IACUC) or other relevant ethics board that reviewed the study protocol, and indicate whether they approved this research or granted a formal waiver of ethical approval<br>• Include an approval number if one was obtained<br>• If the study involved *non-human primates*, add *additional details* about animal welfare and steps taken to ameliorate suffering<br>• If anesthesia, euthanasia, or any kind of animal sacrifice is part of the study, include briefly which substances and/or methods were applied<br><br>**Field Research**<br><br>Include the following details if this study involves the collection of plant, animal, or other materials from a natural setting:<br>• Field permit number<br>• Name of the institution or relevant body that granted permission | |
| **Data Availability**<br><br>Authors are required to make all data underlying the findings described fully available, without restriction, and from the time of publication. PLOS allows rare exceptions to address legal and ethical concerns. See the PLOS Data Policy and FAQ for detailed information. | Yes - all data are fully available without restriction |

| | |
|---|---|
| A Data Availability Statement describing where the data can be found is required at submission. Your answers to this question constitute the Data Availability Statement and **will be published in the article**, if accepted.<br><br>**Important:** Stating 'data available on request from the author' is not sufficient. If your data are only available upon request, select 'No' for the first question and explain your exceptional situation in the text box.<br><br>Do the authors confirm that all data underlying the findings described in their manuscript are fully available without restriction? | |
| **Describe where the data may be found in full sentences. If you are copying our sample text, replace any instances of XXX with the appropriate details.**<br><br>• If the data are **held or will be held in a public repository**, include URLs, accession numbers or DOIs. If this information will only be available after acceptance, indicate this by ticking the box below. For example: *All XXX files are available from the XXX database (accession number(s) XXX, XXX.*).<br>• If the data are all contained **within the manuscript and/or Supporting Information files**, enter the following: *All relevant data are within the manuscript and its Supporting Information files.*<br>• If neither of these applies but you are able to provide **details of access elsewhere**, with or without limitations, please do so. For example:<br><br>*Data cannot be shared publicly because of [XXX]. Data are available from the XXX Institutional Data Access / Ethics Committee (contact via XXX) for researchers who meet the criteria for access to confidential data.*<br><br>*The data underlying the results presented in the study are available from (include the name of the third party* | The sequence reads were deposited in the European Nucleotide Archive with the study accession PRJEB58695 (For ease of retrieval, a full listing of the specific sequences is included in Supplementary table 1). |

| | |
|---|---|
| *and contact information or URL).*<br>• This text is appropriate if the data are owned by a third party and authors do not have permission to share the data.<br><br>* typeset | |
| Additional data availability information: | |

Powered by Editorial Manager® and ProduXion Manager® from Aries Systems Corporation

Click here to access/download;Manuscript;pone-611394.pdf

# Genomic characterization of foodborne *Salmonella enterica* and *Escherichia coli* isolates from Saboba district and Bolgatanga Municipality Ghana

Gabriel Temitope Sunmonu [1], Frederick Adzitey [2], Erkison Ewomazino Odih [1], Boniface Awini Tibile [2], Rejoice Ekli [2], Martin Aduah [2], Anderson O Oaikhena [1], Olabisi C Akinlabi [1], Akebe Luther King Abia [3], Daniel Gyamfi Amoako [3, 4], and Iruka N Okeke [1]

1. Department of Pharmaceutical Microbiology, Faculty of Pharmacy, University of Ibadan, Nigeria.
2. Faculty of Agriculture, Food and Consumer Sciences, University for Development Studies, Tamale, Ghana
3. Antimicrobial Research Unit, College of Health Sciences, University of KwaZulu-Natal, Durban, South Africa
4. Department of Integrative Biology and Bioinformatics, University of Guelph, Ontario, Canada

## Abstract

*Salmonella* and *Escherichia coli* are well-known bacteria commonly associated with foodborne illnesses in humans and animals. Genomic characterization of these pathogens provides valuable insights into their evolution, virulence factors and resistance determinants. This study characterized *Salmonella* (n = 14) and *E. coli* (n = 19) from milk, meat and its associated utensils in Ghana using whole-genome sequencing. Most of the *Salmonella* serovars (Fresno, Plymouth, Infantis, Give and Orleans) identified in this study are yet to be reported in Ghana. The *Salmonella enterica* isolates were largely pan-sensitive but genes conferring resistance to fosfomycin (*fosA7.2*) and tetracycline (*tet(A)*) were detected in one and three isolates respectively. While 7of the *Salmonella* isolates carry the IncI1-I(Gamma) plasmid replicon. While antimicrobial resistance was not common among *Salmonella* strains, most (11/19) of the *E. coli* strains had at least one resistance gene and almost half (8/19) were multidrug resistance and carry mobile elements. Three of the 19 *E. coli* strains belong to serovars commonly associated with enteroaggregative *E. coli* (EAEC) lineage. While strains belonging to virulence-associated lineages lacked key plasmid-encoded virulence plasmids, several plasmid replicons were detected in most of the *E. coli* (14/19) strains. Food contaminated with these pathogens can serve as a vehicle for transmission, posing a significant public health risk and necessitating stringent food safety and hygiene practices to prevent outbreaks. Hence, there is need for continuous surveillance and preventive measures to stop the spread of foodborne pathogens and reduce the risk of associated illnesses in Ghana.

## INTRODUCTION

Milk and meat are essential protein sources that constitute a significant and nutrient-rich component of human diets. However, their consumption is frequently associated with foodborne infections (1), particularly those caused by *Escherichia coli* and *Salmonella* (2-5). Salmonellosis is under-reported in Ghana, and only a few studies have investigated the plausible role of contaminated milk, meat or meat products in facilitating their transmission (6-8). There have been a few reports of meat samples contaminated with *E. coli* in Ghana (9-11). Some of these *E. coli* strains harbour virulence and antimicrobial resistance genes (ARGs), which is of public health concern however few have been characterized in detail.

There are public health and food safety implications of finding E. coli and Salmonella in food because they are invariably of faecal origin. Globally, *Salmonella* and *Escherichia coli* sourced

from milk and meat increasingly exhibit resistance to different classes of antibiotics that is commonly mediated by mobile elements (10, 12-14). The prevalence of multidrug-resistant (MDR) *S. enterica* and *E. coli* is also on the rise in clinical infections (15, 16). Notably, resistance to extended spectrum beta-lactams, trimethoprim/sulfamethoxazole, chloramphenicol and ciprofloxacin has been reported, often associated with plasmids that could mediate their spread, in both *S. enterica* and *E. coli* isolates from milk and retail meats in Ghana (7, 9, 11).

Various methods, including serotyping, antibiotic profiling, pulsed-field gel electrophoresis and whole genome sequencing, have been employed to elucidate the phenotypic and genotypic attributes of foodborne pathogens and determine their interrelationships and connections to pandemic clones of interest (10, 17). Next-generation sequencing (NGS) technology, the most versatile and informative approach, has gained recent prominence (18, 19). NGS is now used by PulseNet to categorize foodborne diseases, enabling nuanced epidemiological investigations (20). Identification of virulence factors, antibiotic resistance genes, and serotypes are all possible by genomic analysis, which can also provide enhanced information on strain inter-relatedness, and therefore enable source attribution. Data on the genomic characterization of *Salmonella* and *E. coli* from milk and meat are few from low- and middle-income countries, including Ghana (6, 21). In light of this gap, this study aims to characterize the resistance, virulence and plasmid profile of *Salmonella* and *E. coli* isolated from fresh different retail meats, milk, and associated samples (handler's hand swab, table, knife and faecal samples) in in Saboba district and Bolgatanga Municipality of Ghana.

## METHODS

### Strains

A total of 33 bacterial isolates (14 *Salmonella* and 19 *E. coli* species) previously isolated from various fresh and ready-to-eat meats, meat sellers' tables, milk, milk-collecting utensils, milkers' hands and faeces of lactating cows were characterized for this study (22, 23). The isolates originated from markets and farms in Bolgatanga Municipality and Saboba District in Northern Ghana and were cryopreserved in 50% glycerol in Luria broth at -80$^{o}$C.

### *Salmonella* and *E. coli* identification

*Salmonella* and *E. coli* were biotyped with the Gram-negative (GN) test kit (Ref: 21341) on VITEK 2 systems (version 2.0, Marcy-l'Etoile, France, Biomérieux) according to manufacturer's instructions. *Salmonella* isolates were additionally confirmed using a latex agglutination kit for *Salmonella* (Oxoid Limited, Basingstoke, UK) and by PCR targeting the *invA* gene as described by Rahn et al. (1992) (24), using PCR oligonucleotides *invA139f* GTGAAATTATCGCCACGTTCGGGCAA and *invA141r* TCATCGCACCGTCAAGGAACC. PCR was performed using PuRe Taq Ready-To-Go PCR Beads (illustra$^{TM}$). The PCR cycle used an initial denaturation temperature of 95$^{0}$C for two minutes, followed by 35 cycles of denaturation at 95$^{0}$C for 30 seconds, annealing at 55$^{0}$C for 30 seconds, extension at 72$^{0}$C for two minutes and then a terminal extension at 72$^{0}$C for five minutes. Visualization of the 284 bp amplicon was accomplished after electrophoresis on 1.5% (w/v) agarose gels stained with Gel red (biotium), using a UVP GelMax transilluminator and imager.

## DNA Extraction, Library Preparation and Whole Genome Sequencing

Genomic DNA of the isolates was extracted using Wizard DNA extraction kit (Promega; Wisconsin, USA) in accordance with manufacturer's protocol. Using a dsDNA Broad Range quantification assay, the extracted DNA was quantified on a Qubit fluorometer (Invitrogen; California, USA). dsDNA libraries were prepared using NEBNext Ultra II FS DNA library kit for Illumina with 96-unique indexes (New England Biolabs, Massachusetts, USA; Cat. No: E6609L). DNA libraries was quantified using dsDNA High Sensitivity quantification assay on a Qubit fluorometer (Invitrogen; California, USA) and fragment length analysed with the Bioanalyzer (Agilent). Denatured libraries were sequenced on an Illumina MiSeq (Illumina, California, USA). The raw sequence reads were *de novo* assembled using SAdes v3.15.3 (25) according to GHRU protocols (https://gitlab.com/cgps/ghru/pipelines/dsl2/pipelines/assembly).

## Sequence Typing of *Salmonella* and *E. coli* Genomes

Sequence reads were deposited in the *Salmonella* and *E. coli* database for *Salmonella* and *E. coli* respectively on EnteroBase (26). Multi-locus sequence types (MLST) for the isolates were determined and core-genome MLST calculated. The *Salmonella* genome assemblies were analysed using the *Salmonella* In-Silico Typing Resource (SISTR) for the prediction of serovars and serogroups (27) (https://github.com/phac-nml/sistr_cmd), while the serotyping of the *E. coli* genome was done using ECtyper (28).

## Identification of AMR, Plasmids and Virulence genes

PlasmidFinder (29) was utilized to identify plasmid replicons that were present in the assembled genomes. AMRFinderPlus v3.10.24 (30) and its associated database (version 2022-04-04.1) were used to predict the antimicrobial resistance genes carried by the isolates and the drug classes to which they probably conferred resistance. Using ARIBA (31) and the virulence factor database (VFDB), we were also able to identify the virulence genes that were present in the isolates.

## Single Nucleotide Polymorphism (SNP) Calling and Phylogenetic analysis

For phylogenetic analysis, reference sequences for the *S. enterica* and *E. coli* genomes were objectively selected from the National Center for Biotechnology Information Reference Sequence (RefSeq) database (https://www.ncbi.nlm.nih.gov/refseq/) using BactinspectorMax v0.1.3 (https://gitlab.com/antunderwood/bactinspector). The selected references were the *S. enterica* subsp. enterica serovar Fresno strain (assembly accession: GCF_003590695.1) and the *E. coli* O25b:H4-ST131 strain (assembly accession: GCF_000285655.3). The sequence reads for each species were then mapped to the chromosome of the reference using BWA (v0.7.17) (32) and variants were called and filtered using bcftools (v1.9) (Danecek et al., 2021) as implemented in the GHRU SNP phylogeny pipeline (https://gitlab.com/cgps/ghru/pipelines/snp_phylogeny). Variant positions were concatenated into a pseudoalignment and used to generate a maximum likelihood tree using iqtree (v1.6.8) (33). SNP distances between the genome pairs were calculated using snp-dists v.0.8.2 (https://github.com/tseemann/snp-dists).

## Data availability

The sequence reads were deposited in the European Nucleotide Archive with the study accession PRJEB58695 (Supplementary table 1).

## RESULTS

### *Salmonella* Serotypes, Sequence Types (STs), Virulence Factors and Phylogeny

We used SISTR software to predict the serovars of the 14 Salmonella strains characterized in this study from whole genome sequence. The most common serotype was Fresno (n=6) followed by Give (n=3), Orleans (n=2), Plymouth (n=1), Agona (n=1) and Infantis (n=1). The *S.* Fresno and *S.* Orleans isolates were from previously unreported sequence types, now designated ST 10742 and ST 10465 respectively (Supplementary table 2). As shown in Figure 1, all the isolates from ready-to-eat pork, mutton and chicken belong to serovar Fresno. *S.* Fresno isolates were also isolated from a meat vendor's knife, as was *S.* Orleans. The three milk isolates, which were all from Saboba, belong to the serovars Plymouth (ST 565), Give (ST 516) and Agona (ST13) (Figure 1). Two more ST 516 *S.* Give isolates were recovered from the faeces of milking cow and from a milking utensil.

Phylogenetic analysis of the 14 *Salmonella enterica* isolates from this study showed that all *S.* Fresno isolates, irrespective of source, clustered together and differed by < 3 SNPs. The two *S.* Orleans isolates were identical (0 SNPs) and the three *S*. Give isolates were also identical, with the isolates originating from milk, faeces of milking cow and from a milking utensil, also in Saboba.

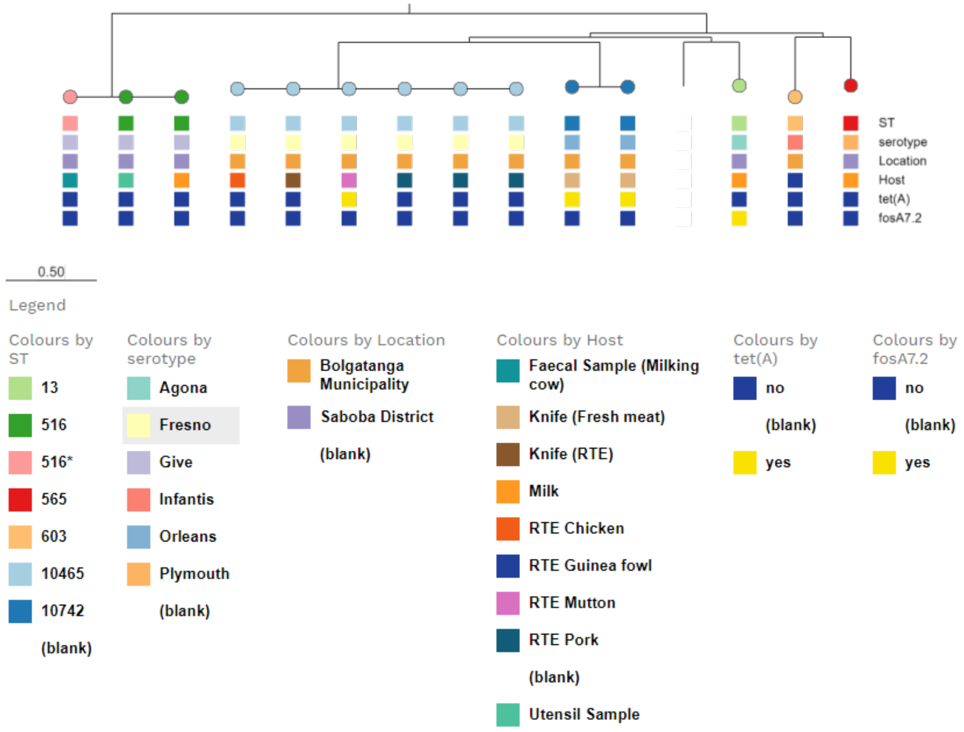


Figure 1: Core genome SNP-based Maximum likelihood tree showing phylogenetic relationships among strains sequenced in this study. The matrix below the tree shows the sequence types, serotypes, location of isolation (Bolgatanga municipality or Saboba district) as well as the source/host sample and presence or absence of the tetA and fosA resistance genes.

All *Salmonella* isolates harboured curli (*csg*) genes as well as *bcf*, *fim* and *ste* fimbrial operons and ten of them, representing all serovars except *S*. Give and Plymouth, carried long polar fimbriae (*lpf)* genes. The *S*. Infantis and *S.* Agona strains carried *ratB* and *shdA*. Type III secretion system effector genes, such as: *inv*, *org*, *prg*, *sif*, *spa*, *sse*, *ssa* and *sop* were detected in all the isolates while *avr* was present in 57.1% (8/14) of the isolates and only one isolate harbours *gogB*. Four of the isolates also encode the cytholethal distending toxin gene, *cdtB* (Supplementary table 3).

**Plasmid Replicons and ARG Profiles of *Salmonella***

The *Salmonella enterica* isolates were largely pan-sensitive but genes conferring resistance to fosfomycin (*fosA7.2*) and tetracycline (*tet(A)*) were detected in one and three isolates respectively. Both *S*. Orleans isolates and one of the *S*. Fresno, from ready-to-eat mutton carried *tetA*, along with an IncI1-I(Gamma) plasmid replicon, which was also seen in six other, *tetA*-negative strains. Interestingly, the IncI1-I(Gamma) plasmid replicon was detected in all isolates from Bolgatanga municipality, irrespective of serovar, and no isolate from Saboba district harboured this plasmid. The fosfomycin resistance gene was found in the *S*. Agona genome, in which no plasmid replicons were detected (Figure 1).

***E. coli* Serotypes, Virulence factors and Phylogeny**

A total of 19 *E. coli* isolates were identified. *E. coli* serotyping using the ECtyper revealed that the most common serotypes among the *E. coli* isolates were -:H7 (n=2), O138:H48 (n=2), O6:H16 (n=2), and O8/O160:H16 (n=2). A number of these serovars and STs are associated with pathogenicity, notably O6:H16 (34), as well as O8/O160:H16 and O77/O17/O44/O106/O73:H18 (ST394; (35-37)). The strains belonging to these lineages lacked the defining virulence genes of the respective pathotypes but did contain accessory virulence genes, as shown in supplementary table 4.

Irrespective of whether they belonged to a lineage commonly associated with virulence, most of the isolates contained a range of adhesins and iron utilization genes. *E. coli* extracellular protein (ECP) export pathway (*ecp/yag*) and *ompA* and type I fimbriae-encoding operon, *fim*, encoding genes seen in most *E. coli* genomes, were present in 94.7% (18/19) of *E. coli* isolates. Fimbriae encoding gene, *f17d*, often seen in enterotoxigenic *E. coli* recovered from animals, was present in the two O8/O160:H16 isolates and an O-:H7 isolate.

The phylogenetic analysis of the 19 *E. coli* isolates from this study and a reference genome (NZ_HG941718.1) based on SNP is presented in Figure 2. The range of isolates was broader than with *Salmonella* but closely related pairs of isolates belonging to the same serovar, and ST were found in three instances. Very similar (2347 SNPs) O6:H16 isolates were recovered from different food preparation table samples in Bolgatanga. One of the two isolates from milk in Saboba belonged to ST2165 and was identical (0 SNPs) to a Saboba ST2165 utensil isolate. The two ST4 isolates from fresh beef and a cow milker's hand differed by 2347 SNPs and are unlikely to be connected.

(a)

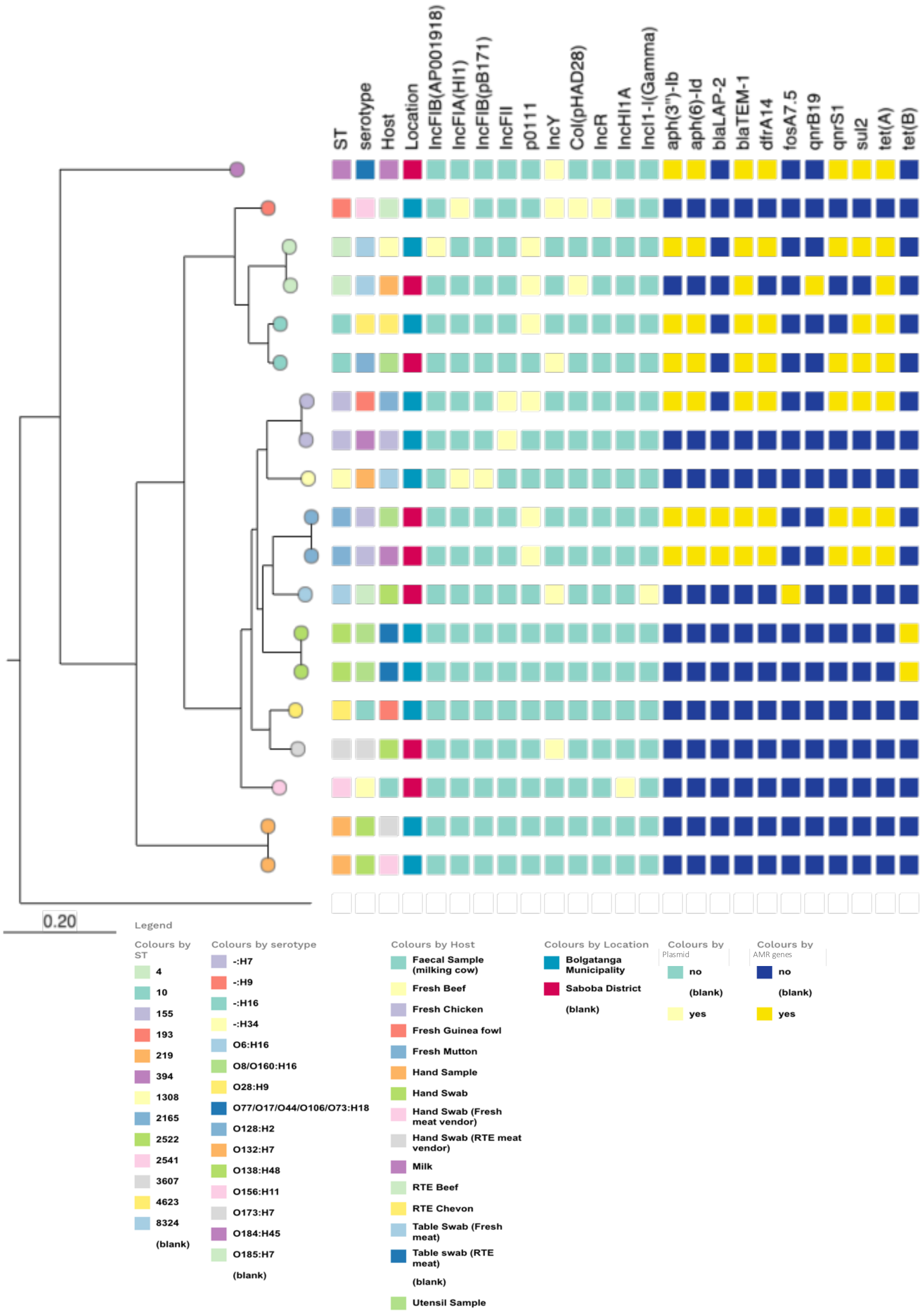

ST
serotype
Host
Location
IncFIB(AP001918)
IncFIA(HI1)
IncFIB(pB171)
IncFII
p0111
IncY
Col(pHAD28)
IncR
IncHI1A
IncI1-I(Gamma)
aph(3'')-Ib
aph(6)-Id
blaLAP-2
blaTEM-1
dfrA14
fosA7.5
qnrB19
qnrS1
sul2
tet(A)
tet(B)
0.20
Legend
Colours by ST
4
10
155
193
219
394
1308
2165
2522
2541
3607
4623
8324
(blank)
Colours by serotype
-:H7
-:H9
-:H16
-:H34
O6:H16
O8/O160:H16
O28:H9
O77/O17/O44/O106/O73:H18
O128:H2
O132:H7
O138:H48
O156:H11
O173:H7
O184:H45
O185:H7
(blank)
Colours by Host
Faecal Sample (milking cow)
Fresh Beef
Fresh Chicken
Fresh Guinea fowl
Fresh Mutton
Hand Sample
Hand Swab
Hand Swab (Fresh meat vendor)
Hand Swab (RTE meat vendor)
Milk
RTE Beef
RTE Chevon
Table Swab (Fresh meat)
Table swab (RTE meat)
(blank)
Utensil Sample
Colours by Location
Bolgatanga Municipality
Saboba District
(blank)
Colours by Plasmid
no
(blank)
yes
Colours by AMR genes
no
(blank)
yes


(b)

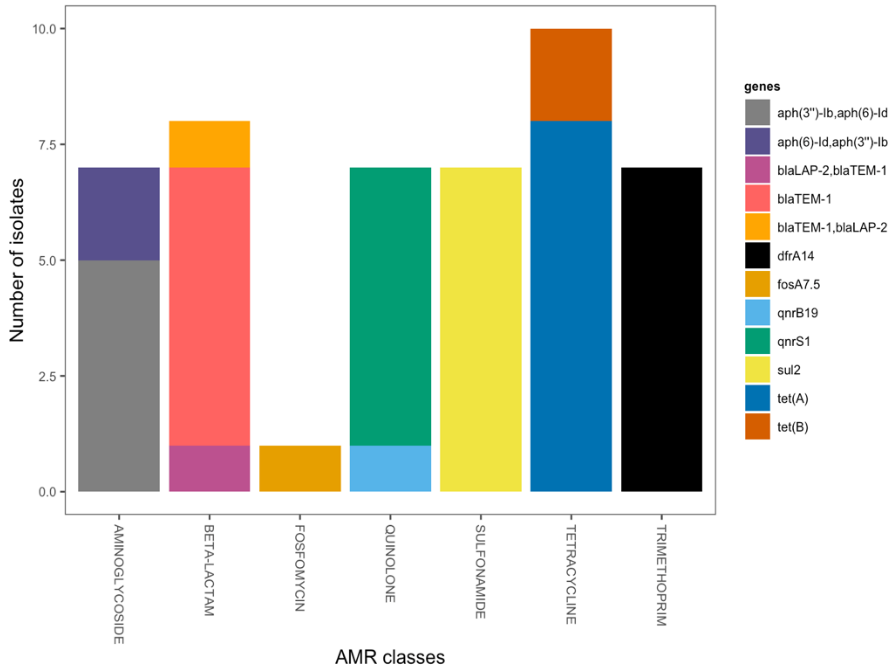


Figure 2: Phylogeny, plasmid replicons and resistance genes of *E. coli* isolates. A. SNP-based phylogenetic tree of the *E. coli* isolates showing the range of sequence types, serovars, plasmid replicons and resistance genes detected (b) AMR determinants detected in *E. coli* and the resistance they confer

**Plasmid Replicons and ARG Profiles of *E. coli***

Antimicrobial resistance determinants present in the *E. coli* isolates include those encoding resistance to aminoglycosides (*aph(3")-Ib*, *aph(6)-Id*, *aph(6)-Id*, *aph(3")-Ib*), beta-lactams (*bla*$_{LAP-2}$, *bla*$_{TEM-1}$), fosfomycin (*fosA7.5*), quinolones (*qnrB19*, *qnrS1*), sulfonamide (*sul2*), tetracycline (*tet(A)*, *tet(B)*) and trimethoprim (*dfrA14*) (Figure 2). At least 3 antimicrobial resistant genes (ARGs) which confer resistance to different classes of antibiotics were present in 8 isolates. Three isolates carried one ARG each while 8 isolates had no ARGs. Six strains carried the genes *aph(3'')-Ib, aph(6)-Id, blaTEM-1, dfrA14, sul2, qnrS1* and *tet(A).* The *dfrA14-qnrS1-tet(A) resistance* gene combination has previously been reported from Nigeria, being part of a transposon transmitted in an IncX plasmid (38). In this study however, IncX replicons were not detected. The most common plasmid replicon type detected among the *E. coli* isolates was pO111 (n=6), originally described in an *E. coli* virulence plasmid and found in the aforementioned strains belonging to virulence-associated lineages. The other plasmid replicon types detected were IncY (n=5), IncFII (n=2), IncFIA(HI1) (n=2), Col(pHAD28) (n=2), IncFIB(pB171) (n=1), IncR (n=1), IncHI1A (n=1) and IncI1-I(Gamma), which was common among the *Salmonella*, (n=1) (Figure 2). All multidrug resistant strains encode pO111 or IncY replicons.

**DISCUSSION**

*Salmonella* and *E. coli* are the main causes of bacterial foodborne illnesses in Ghana (39). Retail meat, along with milk and their products are recognized as primary sources of foodborne Salmonellosis (9) and *E. coli* infection (6). Post-cooking handling practices, exposure during the

points of sale, and suboptimal meat storage conditions collectively contribute to an increased presence of both pathogenic and spoilage bacteria in ready-to-eat (RTE) meat (40). In this study, we characterized the genomes of 14 *Salmonella* and 19 *E. coli* previously isolated from Saboba district and Bolgatanga Municipality in northern Ghana.

The *Salmonella* serovars (Fresno, Plymouth, Infantis, Give and Orleans) identified in this study are yet to be reported in Ghana, but Guinee et al. (1961) (41) isolated *S.* Agona from cattle in Ghana. Isolation of *S.* Infantis from retail poultry meat has also been reported in Ecuador (42), Belgium (43) and Italy (44) and all the serovars detected in this study have been implicated in human infections. The identification of Salmonella serovars not previously documented in the country in meat and milk products highlights the need for heightened surveillance and preventive measures to curb the spread of foodborne pathogens and reduce the risk of associated illnesses.

Unlike *Salmonella*, not all *E. coli* are potential pathogens. However, *E. coli* serve as markers for faecal contamination and therefore the potential that other pathogens are present. The predominant *E. coli* STs (ST4, ST10, ST219, ST2522) detected in our study have been previously isolated from food animals and have been associated with pathogenicity (45-47). While none of the isolates carried genes encoding ETEC heat-sensitive or heat-labile enterotoxins, f17d fimbrial genes present in four of the *E. coli* genomes encode ETEC colonization factors commonly associated with colonization of cattle and other ruminant isolates (48). *E. coli,* F17 fimbriae are associated with pathogenic *E. coli* recovered from diarrhoea and septicaemia outbreaks in calves, lambs, and humans, including from outbreaks. Additionally, two of the ST4 *E. coli* isolates from this study not harbouring f17d fimbrial genes belong to the serovar O6:H16, one of the most widely disseminated lineages of human enterotoxigenic *E. coli* (ETEC). O6:H16 ETEC cause outbreaks, often associated with food and/or inadequate handwashing (49-51). ETEC, by definition, produce plasmid-encoded heat-labile and/or heat stable toxins not present in the genomes of the isolates from this study. However, the serovars O8/O160:H16 and O77/O17/O44/O106/O73:H18 belong to a previously described enteroaggregative *E. coli* (EAEC) lineage (35, 37). Isolates from this study belonging to the serovars O8/O160:H16 and O77/O17/O44/O106/O73:H18 possess no EAEC accessory genes. These strains are from virulent lineages but lack key virulence genes that are plasmid-encoded, which could mean that these plasmids were lost in the food chain or during isolation but could be reacquired. Nevertheless, the presence of these strains in food could increase the risk of foodborne illness.

While strains belonging to virulence-associated lineages lacked key plasmid-encoded virulence plasmids, several plasmid replicons were detected in the isolate genomes. ColE, IncI1, IncF, and IncX were commonly detected plasmid replicons in *Salmonella* from food animals in the US according to McMillan et al. (2019) (52). In this study, the IncI1 replicon was predominant, and nine of the thirteen *Salmonella* strains harboured IncI1 plasmid replicon, three harboured the *tetA*. This is likely to be an instance of a successful mobile element with extraordinary local reach, a few of which have been reported from West Africa, including Ghana, in the past (38, 53-55). The IncI1-I(Gamma) plasmid replicon observed among *S. enterica* strains was detected in all *Salmonella* isolates from Bolgatanga municipality - three different serovars - and none of the Saboba district isolates. However, one *E. coli* isolate (from a recently reported ST, ST8274) from

Saboba did have this replicon. As it is an IncI1 plasmid replicon, its plasmid should be better characterized and, it should remain under surveillance because numerous articles have reported associations with multiple ARGs, such as *tetB*, *tetAR*, $bla_{CMY-2}$, $bla_{TEM-1}$, *aac3VIa*, *aphA*, *aadA* and *sul1* (52), $bla_{CTXM-1}$ (56), *strA*, *strB*, *cmlA*, *floR*, $bla_{SHV-12}$, $bla_{OXA-2}$ and *FosA3* (57) in IncI1 plasmids in *Salmonella*. As our own sequence was generated by short read only, the first step would be to generate long read sequence that could fully assemble the plasmid and make it possible to identify genetic factors supporting its success.

Among the *E. coli* isolates, the plasmid replicons pO111 was the most common replicon. A previous study by Balbuena-Alonso et al. (2022) (58), revealed that pO111 is usually associated with extended spectrum beta lactamases gene and is very common in food and clinical isolates. In this study, all isolates carrying pO111 harbour at least one beta-lactamase. Likewise, all the pO111 plasmid bearing isolates in this study carry ARGs that confer resistance to at least 4 classes of antibiotics. Altogether, these data demonstrate a concerning reservoir of resistance genes in these foodborne bacteria.

**Conclusion**

This study has characterized the genomes of *Salmonella* and *E. coli* in milk, meat and their associated utensils. The diverse serovars and virulence genes detected in *Salmonella* strains indicate potential pathogenicity. All *E. coli* are not always pathogenic, but they serve as indicators of faecal contamination, suggesting a potential risk for the presence of other pathogens. The presence of EAEC lineages in food is concerning as these bacteria are known to cause diarrhoea, especially in children and immunocompromised individuals. While antimicrobial resistance was not common among *Salmonella* strains, most of the *E. coli* strain had at least one resistance gene and almost half were multidrug resistance and carry mobile elements. Food contaminated with these pathogens can serve as a vehicle for transmission, posing a significant public health risk and necessitating stringent food safety and hygiene practices to prevent outbreaks. Hence, there is need for continuous surveillance and preventive measures to stop the spread of foodborne pathogens and reduce the risk of associated illnesses in Ghana.

**Supplementary Materials**

Supplementary Table 1: Accession numbers for genomes generated in this study

Supplementary Table 2: Novel *Salmonella* allelic profile and assigned ST.

Supplementary Table 3: *Salmonella* strain metadata including serotype, ST, plasmid replicon, AMR and virulence profile.

Supplementary Table 4: *E. coli* strain metadata including serotype, ST, plasmid replicon, AMR and virulence profile.

**Acknowledgements**

We thank Ayorinde Afolayan, Faith I. Oni, and Abeeb Adeniyi for technical assistance and Jola-Ade Ajiboye, Kesiena Akpede and Pernille Nilsson for logistic support.

Whole genome sequencing and analysis for this project was supported through SEQAFRICA. The SEQAFRICA project is funded by the Department of Health and Social Care's Fleming Fund using

UK aid. The views expressed in this publication are those of the authors and not necessarily those of the UK Department of Health and Social Care or its Management Agent, Mott MacDonald. INO is a Calestous Juma Fellow supported by the Bill and Melinda Gates Foundation.

**REFERENCES:**


1. Grace D, Wu F, Havelaar AH. MILK Symposium review: Foodborne diseases from milk and milk products in developing countries-Review of causes and health and economic implications. J Dairy Sci. 2020;103(11):9715-29.
2. Abraham S, O'Dea M, Sahibzada S, Hewson K, Pavic A, Veltman T, et al. Escherichia coli and Salmonella spp. isolated from Australian meat chickens remain susceptible to critically important antimicrobial agents. PLoS One. 2019;14(10):e0224281.
3. Elzhraa F, Al-Ashmawy M, El-Sherbini M, Abdelkhalek A. Critical occurrence of verotoxgenic E.coli and non-typhoidal salmonella in some heat treated dairy products. Ital J Food Saf. 2021;10(2):9318.
4. Gutema FD, Abdi RD, Agga GE, Firew S, Rasschaert G, Mattheus W, et al. Assessment of beef carcass contamination with Salmonella and E. coli O 157 in slaughterhouses in Bishoftu, Ethiopia. International Journal of Food Contamination. 2021;8:1-9.
5. Lay KK, Jeamsripong S, Sunn KP, Angkititrakul S, Prathan R, Srisanga S, et al. Colistin Resistance and ESBL Production in Salmonella and Escherichia coli from Pigs and Pork in the Thailand, Cambodia, Lao PDR, and Myanmar Border Area. Antibiotics (Basel). 2021;10(6).
6. Adzitey F, Asante J, Kumalo HM, Khan RB, Somboro AM, Amoako DG. Genomic Investigation into the Virulome, Pathogenicity, Stress Response Factors, Clonal Lineages, and Phylogenetic Relationship of Escherichia coli Strains Isolated from Meat Sources in Ghana. Genes (Basel). 2020;11(12).
7. Parry-Hanson Kunadu A, Holmes M, Miller EL, Grant AJ. Microbiological quality and antimicrobial resistance characterization of Salmonella spp. in fresh milk value chains in Ghana. Int J Food Microbiol. 2018;277:41-9.
8. Tay MYF, Adzitey F, Sultan SA, Tati JM, Seow KLG, Schlundt J. Whole-Genome Sequencing of Nontyphoidal Salmonella enterica Isolates Obtained from Various Meat Types in Ghana. Microbiol Resour Announc. 2019;8(15).
9. Adzitey F, Assoah-Peprah P, Teye GA. Whole-genome sequencing of Escherichia coli isolated from contaminated meat samples collected from the Northern Region of Ghana reveals the presence of multiple antimicrobial resistance genes. J Glob Antimicrob Resist. 2019;18:179-82.
10. Adzitey F, Teye GA, Amoako DG. Prevalence, phylogenomic insights, and phenotypic characterization of Salmonella enterica isolated from meats in the Tamale metropolis of Ghana. Food Sci Nutr. 2020;8(7):3647-55.
11. Falgenhauer L, Imirzalioglu C, Oppong K, Akenten CW, Hogan B, Krumkamp R, et al. Detection and Characterization of ESBL-Producing Escherichia coli From Humans and Poultry in Ghana. Front Microbiol. 2018;9:3358.
12. Moawad AA, Hotzel H, Awad O, Tomaso H, Neubauer H, Hafez HM, et al. Occurrence of Salmonella enterica and Escherichia coli in raw chicken and beef meat in northern Egypt and dissemination of their antibiotic resistance markers. Gut Pathog. 2017;9:57.
13. Geletu US, Usmael MA, Ibrahim AM. Isolation, Identification, and Susceptibility Profile of E. coli, Salmonella, and S. aureus in Dairy Farm and Their Public Health Implication in Central Ethiopia. Vet Med Int. 2022;2022:1887977.

14. Worku W, Desta M, Menjetta T. High prevalence and antimicrobial susceptibility pattern of salmonella species and extended-spectrum beta-lactamase producing Escherichia coli from raw cattle meat at butcher houses in Hawassa city, Sidama regional state, Ethiopia. PLoS One. 2022;17(1):e0262308.
15. Lyu N, Feng Y, Pan Y, Huang H, Liu Y, Xue C, et al. Genomic Characterization of Salmonella enterica Isolates From Retail Meat in Beijing, China. Front Microbiol. 2021;12:636332.
16. Zhan Z, Xu X, Gu Z, Meng J, Wufuer X, Wang M, et al. Molecular epidemiology and antimicrobial resistance of invasive non-typhoidal Salmonella in China, 2007-2016. Infect Drug Resist. 2019;12:2885-97.
17. Jaja IF, Bhembe NL, Green E, Oguttu J, Muchenje V. Molecular characterisation of antibiotic-resistant Salmonella enterica isolates recovered from meat in South Africa. Acta Trop. 2019;190:129-36.
18. Rantsiou K, Kathariou S, Winkler A, Skandamis P, Saint-Cyr MJ, Rouzeau-Szynalski K, et al. Next generation microbiological risk assessment: opportunities of whole genome sequencing (WGS) for foodborne pathogen surveillance, source tracking and risk assessment. Int J Food Microbiol. 2018;287:3-9.
19. Hilt EE, Ferrieri P. Next Generation and Other Sequencing Technologies in Diagnostic Microbiology and Infectious Diseases. Genes (Basel). 2022;13(9).
20. Nadon C, Van Walle I, Gerner-Smidt P, Campos J, Chinen I, Concepcion-Acevedo J, et al. PulseNet International: Vision for the implementation of whole genome sequencing (WGS) for global food-borne disease surveillance. Euro Surveill. 2017;22(23).
21. Saba CK, Escudero JA, Herrera-Leon S, Porrero MC, Suarez M, Dominguez L, et al. First identification of Salmonella Urbana and Salmonella Ouakam in humans in Africa. J Infect Dev Ctries. 2013;7(10):691-5.
22. Abass A, Adzitey F, Huda N. Escherichia coli of Ready-to-Eat (RTE) Meats Origin Showed Resistance to Antibiotics Used by Farmers. Antibiotics (Basel). 2020;9(12).
23. Aduah M, Adzitey F, Amoako DG, Abia ALK, Ekli R, Teye GA, et al. Not All Street Food Is Bad: Low Prevalence of Antibiotic-Resistant Salmonella enterica in Ready-to-Eat (RTE) Meats in Ghana Is Associated with Good Vendors' Knowledge of Meat Safety. Foods. 2021;10(5).
24. Rahn K, De Grandis SA, Clarke RC, McEwen SA, Galan JE, Ginocchio C, et al. Amplification of an invA gene sequence of Salmonella typhimurium by polymerase chain reaction as a specific method of detection of Salmonella. Mol Cell Probes. 1992;6(4):271-9.
25. Prjibelski A, Antipov D, Meleshko D, Lapidus A, Korobeynikov A. Using SPAdes De Novo Assembler. Curr Protoc Bioinformatics. 2020;70(1):e102.
26. Zhou Z, Alikhan NF, Mohamed K, Fan Y, Agama Study G, Achtman M. The EnteroBase user's guide, with case studies on Salmonella transmissions, Yersinia pestis phylogeny, and Escherichia core genomic diversity. Genome Res. 2020;30(1):138-52.
27. Yoshida CE, Kruczkiewicz P, Laing CR, Lingohr EJ, Gannon VP, Nash JH, et al. The Salmonella In Silico Typing Resource (SISTR): An Open Web-Accessible Tool for Rapidly Typing and Subtyping Draft Salmonella Genome Assemblies. PLoS One. 2016;11(1):e0147101.

28. Bessonov K, Laing C, Robertson J, Yong I, Ziebell K, Gannon VPJ, et al. ECTyper: in silico Escherichia coli serotype and species prediction from raw and assembled whole-genome sequence data. Microb Genom. 2021;7(12).
29. Carattoli A, Zankari E, Garcia-Fernandez A, Voldby Larsen M, Lund O, Villa L, et al. In silico detection and typing of plasmids using PlasmidFinder and plasmid multilocus sequence typing. Antimicrob Agents Chemother. 2014;58(7):3895-903.
30. Feldgarden M, Brover V, Gonzalez-Escalona N, Frye JG, Haendiges J, Haft DH, et al. AMRFinderPlus and the Reference Gene Catalog facilitate examination of the genomic links among antimicrobial resistance, stress response, and virulence. Sci Rep. 2021;11(1):12728.
31. Hunt M, Mather AE, Sanchez-Buso L, Page AJ, Parkhill J, Keane JA, et al. ARIBA: rapid antimicrobial resistance genotyping directly from sequencing reads. Microb Genom. 2017;3(10):e000131.
32. Li H, Durbin R. Fast and accurate short read alignment with Burrows-Wheeler transform. Bioinformatics. 2009;25(14):1754-60.
33. Nguyen LT, Schmidt HA, von Haeseler A, Minh BQ. IQ-TREE: a fast and effective stochastic algorithm for estimating maximum-likelihood phylogenies. Mol Biol Evol. 2015;32(1):268-74.
34. Scotland SM, Gross RJ, Rowe B. Serotype-related enterotoxigenicity in Escherichia coli O6.H16 and O148.H28. J Hyg (Lond). 1977;79(3):395-403.
35. Okeke IN, Wallace-Gadsden F, Simons HR, Matthews N, Labar AS, Hwang J, et al. Multi-locus sequence typing of enteroaggregative Escherichia coli isolates from Nigerian children uncovers multiple lineages. PLoS One. 2010;5(11):e14093.
36. Chattaway MA, Day M, Mtwale J, White E, Rogers J, Day M, et al. Clonality, virulence and antimicrobial resistance of enteroaggregative Escherichia coli from Mirzapur, Bangladesh. J Med Microbiol. 2017;66(10):1429-35.
37. Chen Y, Chen X, Zheng S, Yu F, Kong H, Yang Q, et al. Serotypes, genotypes and antimicrobial resistance patterns of human diarrhoeagenic Escherichia coli isolates circulating in southeastern China. Clin Microbiol Infect. 2014;20(1):52-8.
38. Sumrall ET, Gallo EB, Aboderin AO, Lamikanra A, Okeke IN. Dissemination of the transmissible quinolone-resistance gene qnrS1 by IncX plasmids in Nigeria. PLoS One. 2014;9(10):e110279.
39. Christiana Cudjoe D, Balali GI, Titus OO, Osafo R, Taufiq M. Food Safety in Sub-Sahara Africa, An insight into Ghana and Nigeria. Environ Health Insights. 2022;16:11786302221142484.
40. Henriques A, Fraqueza M. Listeria monocytogenes and ready-to-eat meat-based food products: Incidence and control. Listeria monocytogenes: incidence, growth behavior and control. 2015:71-103.
41. Guinee PA, Kampelmacher EH, Willems HM. Six new Salmonella types, isolated in Ghana (S. volta, S. agona, S. wa, S. techimani, S. mampong and S. tafo). Antonie Van Leeuwenhoek. 1961;27:469-72.
42. Mejia L, Vela G, Zapata S. High Occurrence of Multiresistant Salmonella Infantis in Retail Meat in Ecuador. Foodborne Pathog Dis. 2021;18(1):41-8.
43. Zeng H, De Reu K, Gabriel S, Mattheus W, De Zutter L, Rasschaert G. Salmonella prevalence and persistence in industrialized poultry slaughterhouses. Poult Sci. 2021;100(4):100991.

44. Casagrande Proietti P, Musa L, Stefanetti V, Orsini M, Toppi V, Branciari R, et al. mcr-1-Mediated Colistin Resistance and Genomic Characterization of Antimicrobial Resistance in ESBL-Producing Salmonella Infantis Strains from a Broiler Meat Production Chain in Italy. Antibiotics (Basel). 2022;11(6).
45. Araujo S, I ATS, Tacao M, Patinha C, Alves A, Henriques I. Characterization of antibiotic resistant and pathogenic Escherichia coli in irrigation water and vegetables in household farms. Int J Food Microbiol. 2017;257:192-200.
46. Silva A, Silva V, Pereira JE, Maltez L, Igrejas G, Valentao P, et al. Antimicrobial Resistance and Clonal Lineages of Escherichia coli from Food-Producing Animals. Antibiotics (Basel). 2023;12(6).
47. Zhao X, Miao Y, Adam FEA, Zhao H, Zhou Z, Su M, et al. ESBLs-producing Escherichia coli from sheep-origin: Genome-wide virulence genes identification and in vivo virulence assessment in mice and Galleria mellonella. Transbound Emerg Dis. 2022;69(6):3606-17.
48. Bihannic M, Ghanbarpour R, Auvray F, Cavalie L, Chatre P, Boury M, et al. Identification and detection of three new F17 fimbrial variants in Escherichia coli strains isolated from cattle. Vet Res. 2014;45(1):76.
49. Pacheco AB, Soares KC, de Almeida DF, Viboud GI, Binsztein N, Ferreira LC. Clonal nature of enterotoxigenic Escherichia coli serotype O6: H16 revealed by randomly amplified polymorphic DNA analysis. Journal of clinical microbiology. 1998;36(7):2099-102.
50. Beatty ME, Adcock PM, Smith SW, Quinlan K, Kamimoto LA, Rowe SY, et al. Epidemic diarrhea due to enterotoxigenic Escherichia coli. Clin Infect Dis. 2006;42(3):329-34.
51. Willshaw GA, Cheasty T, Rowe B, Smith HR, Faithfull-Davies DN, Brooks TG. Isolation of enterotoxigenic Escherichia coli from British troops in Saudi Arabia. Epidemiol Infect. 1995;115(3):455-63.
52. McMillan EA, Gupta SK, Williams LE, Jove T, Hiott LM, Woodley TA, et al. Antimicrobial Resistance Genes, Cassettes, and Plasmids Present in Salmonella enterica Associated With United States Food Animals. Front Microbiol. 2019;10:832.
53. Dekker D, Eibach D, Boahen KG, Akenten CW, Pfeifer Y, Zautner AE, et al. Fluoroquinolone-Resistant Salmonella enterica, Campylobacter spp., and Arcobacter butzleri from Local and Imported Poultry Meat in Kumasi, Ghana. Foodborne Pathog Dis. 2019;16(5):352-8.
54. Labar AS, Millman JS, Ruebush E, Opintan JA, Bishar RA, Aboderin AO, et al. Regional dissemination of a trimethoprim-resistance gene cassette via a successful transposable element. PLoS One. 2012;7(5):e38142.
55. Monarrez R, Braun M, Coburn-Flynn O, Botelho J, Odetoyin BW, Otero-Vera JI, et al. A large self-transmissible resistance plasmid from Nigeria contains genes that ameliorate a carrying cost. Sci Rep. 2019;9(1):19624.
56. Abraham S, Kirkwood RN, Laird T, Saputra S, Mitchell T, Singh M, et al. Dissemination and persistence of extended-spectrum cephalosporin-resistance encoding IncI1-bla(CTXM-1) plasmid among Escherichia coli in pigs. ISME J. 2018;12(10):2352-62.

57. Foley SL, Kaldhone PR, Ricke SC, Han J. Incompatibility Group I1 (IncI1) Plasmids: Their Genetics, Biology, and Public Health Relevance. Microbiol Mol Biol Rev. 2021;85(2).
58. Balbuena-Alonso MG, Cortes-Cortes G, Kim JW, Lozano-Zarain P, Camps M, Del Carmen Rocha-Gracia R. Genomic analysis of plasmid content in food isolates of E. coli strongly supports its role as a reservoir for the horizontal transfer of virulence and antibiotic resistance genes. Plasmid. 2022;123-124:102650.

Click here to access/download
**Supporting Information**
supp-1.xlsx

Click here to access/download
**Supporting Information**
supp-2.xlsx

Click here to access/download
**Supporting Information**
supp-3.xlsx

Click here to access/download
**Supporting Information**
supp-4.xlsx

Click here to access/download
**Supporting Information**
msfile-1.pdf
