# Supplementary material for: Genomic characterization of foodborne Salmonella enterica and Escherichia coli isolates from Saboba district and Bolgatanga Municipality Ghana
Source: PLoS One. 2025 Feb 7;20(2):e0315583. doi: 10.1371/journal.pone.0315583 (PMC11805442; doi:10.1371/journal.pone.0315583)
Supplement: Attachment — Submitted filename: Response to Reviewer.docx [file pone.0315583.s008.docx]

Reviewer #4:

• Line 25: Authors must understand that research publication is a reported work done hence all verbs must be in the past tense. Hence “belong” must be “belonged” and “carrying” be “carried”. The manuscript is rife with such errors and must be corrected

Author’s response: The reviewer is correct that the work was done in the past, but the strains still carry the plasmids mentioned in the paper, which is why past tense was not used. However to satisfy the reviewer, we have changed this in line 22 and 25

• Line 25: I again ask the authors to define which mobile elements as there are several. If it is a plasmid, then authors must state it.

Author’s response: We have revised this statement in line 25.

• Line 98-99: This implies that apart from the VITEK no initial confirmation was done for the E. coli strains like Salmonella. Any reason for that?

Author’s response: All the isolates were ultimately sequenced, which is the best confirmation we could have had. We used other methods to preview isolates for sequencing. E. coli is relatively simple to assure using basic cultural methods. Salmonella, a bit more challenging, which is why we used VITEK.

• Line 128: Please provide a reference or link for the VFDB platform or publication

Author’s response: We have added the link for the VFDB in line 128

• Line 183: Same comment as Line 25

Author’s response: We have implemented this in lines 161, 163 and 183 as recommended.

• Line 190: The “,” between “other” and “tetA” is not needed.

Author’s response: We have removed the “,” as suggested in line 190.

• Line 191-192: The plasmid information is conspicuously missing in the Figure 1

Author’s response: We have reconstructed figure 1 to show the plasmid information in line 173 and 177.

• Line 196: I suggest the addition of sequence type to the title like in Salmonella

Author’s response: We have added this in line 196.

• Line 294- 296: The sentence is not reading right. Authors must rephrase it

Author’s response: We have rephrased this as captured in line 293-297

• Line 303: It is suggested to authors to be more specific by writing “ association of the Inc1 plasmid replicon

Author’s response: We have rephrased this as captured in line 304

• Line 313: Same comment as in Line 25

Author’s response: We have implemented this in line 314 and 327

• Line 326: Should read “multidrug-resistant”

Author’s response: We have implemented this in line 327

• Line 330-331: The sentence is not reading right

Author’s response: We added a comma after regulations in line 330 and changed “them” to “regulations” for a smooth read.

• Line 333: Saying “elsewhere in West Africa” is vague. Rather authors should say “and by extension West Africa”.

Author’s response: We have implemented this in line 333 and 334
